# Soil Inoculation and Blocker-Mediated Sequencing Show Effects of the Antibacterial T6SS on Agrobacterial Tumorigenesis and Gallobiome

**DOI:** 10.1128/mbio.00177-23

**Published:** 2023-03-06

**Authors:** Si-Chong Wang, Ai-Ping Chen, Shu-Jen Chou, Chih-Horng Kuo, Erh-Min Lai

**Affiliations:** a Institute of Plant and Microbial Biology, Academia Sinica, Taipei, Taiwan; b Department of Life Science, National Central University, Taoyuan, Taiwan; Hebrew University of Jerusalem; University of Toronto

**Keywords:** *Agrobacterium tumefaciens*, crown gall, type VI secretion system, microbiota, 16S rRNA gene amplicon sequencing

## Abstract

The type VI secretion system (T6SS) is deployed by many proteobacteria to secrete effector proteins into bacterial competitors for competition or eukaryotic cells for pathogenesis. Agrobacteria, a group of soilborne phytopathogens causing crown gall disease on various plant species, deploy the T6SS to attack closely and distantly related bacterial species *in vitro* and *in planta*. Current evidence suggests that the T6SS is not essential for pathogenesis under direct inoculation, but it remains unknown whether the T6SS influences natural disease incidence or the microbial community within crown galls (i.e., the gallobiome). To address these two key questions, we established a soil inoculation method on wounded tomato seedlings that mimics natural infections and developed a bacterial 16S rRNA gene amplicon enrichment sequencing platform. By comparing the *Agrobacterium* wild-type strain C58 with two T6SS mutants, we demonstrate that the T6SS influences both disease occurrence and gallobiome composition. Based on multiple inoculation trials across seasons, all three strains induced tumors, but the mutants had significantly lower disease incidences. The season of inoculation played a more important role than the T6SS in shaping the gallobiome. The influence of the T6SS was evident in summer, during which two *Sphingomonadaceae* species and the family *Burkholderiaceae* were enriched in the gallobiome induced by the mutants. Further *in vitro* competition and colonization assays demonstrated the T6SS-mediated antagonism to a *Sphingomonas* sp. R1 strain isolated from tomato rhizosphere in this study. In conclusion, this work demonstrates that the *Agrobacterium* T6SS promotes tumorigenesis in infection processes and provides competitive advantages in gall-associated microbiota.

## INTRODUCTION

Many proteobacteria, including pathogens and commensals, deploy the type VI secretion system (T6SS) for antagonism or pathogenesis (1, 2) . The T6SS is a protein translocation apparatus used to inject effectors into target cells, mainly in a contact-dependent manner. Based on the destination and biological function of known effectors (3), the T6SS mainly functions as an antibacterial weapon used by bacteria to inhibit or kill the competing bacterial species, thus providing a competitive advantage and shaping microbiota in their ecological niche (4).

Previous studies of the microbiome associated with animal guts or plants indicated that the T6SS genes are enriched in these communities, suggesting the T6SS may be important for niche competition (5–8). Metagenomic analysis of human gut microbiota revealed a role of the T6SS in the domination of the gut symbiont Bacteroides fragilis by targeting other members of the microbiome *in vitro* and *in vivo* (6, 7). A recent study further showed that a murine pathogen, Citrobacter rodentium, and resident commensal *Enterobacteriaceae* share the same strategy by using the T6SS for niche competition in the murine gastrointestinal tract (9). The T6SS is also deployed by plant pathogens to gain a competitive growth advantage *in planta* as well as for beneficial bacteria to prevent or reduce disease symptoms caused by competing pathogens (10–12). Comparative metagenomic analysis of microbiota between T6SS^+^ and T6SS^–^ bacterial strains were also carried out. The gut microbiota of pests infected by Pseudomonas protegens, a plant-beneficial bacterium capable of invading insect pests, showed that the T6SS has no significant impact on microbiota diversity at phylum/class level but affects the abundance of *Enterobacteriaceae* (13). These studies collectively suggest that the T6SS is a potent antibacterial weapon used by invading pathogens or resident bacteria to gain competitive advantage in their ecological niches. However, knowledge regarding the degree to which the T6SS shapes the microbiota and the molecular mechanisms of interbacterial competition in complex microbial communities are limited.

Agrobacteria are a diverse group of bacteria that include members from several genera (14). These plant pathogens are capable of inducing crown gall or hairy root disease on plants by transferring a piece of DNA named transfer DNA (T-DNA) from bacteria into plants via the type IV secretion system (T4SS) (15). Another secretion system, the T6SS is highly conserved in several *Agrobacterium* species and plays a role in interbacterial competition (16–18). Among these *Agrobacterium* species, the T6SS is encoded by a gene cluster consisting of an *imp* operon encoding the main T6SS components and an *hcp* operon encoding the puncturing device and effectors (10, 18, 19). Using a key *Agrobacterium* reference strain, C58, which is commonly known as a member of A. tumefaciens but recently reclassified as A. fabrum (20), we previously discovered that it uses a T6SS DNase effector to gain competitive growth advantage *in vitro* and *in planta* (10). Interestingly, agrobacteria with incompatible effector-immunity (EI) pairs exhibit strong antagonism between species, while only weak or nondetectable effects within species (16). Moreover, a higher T6SS-mediated killing outcome was observed when nutrients were scarce, as opposed to nutrient-rich conditions (21). Thus, genetic and environmental factors beyond EI pairs also contribute to interbacterial competition.

To date, agrobacterial T6SS has been only demonstrated as an antibacterial weapon (16–18). There is no evidence for its role in promoting virulence when tumor assays were conducted in sterile conditions or when it was directly inoculated on the stems of various plant species, including tomato plants (19). Considering that agrobacteria may need to compete with other bacteria in bulk soil or rhizosphere to gain access to plant wounds for inciting crown galls, we reasoned that agrobacterial T6SS may influence the microbial community and pathogenesis under a more natural setting. To address these questions, we developed a soil inoculation protocol to mimic natural infection on wounded tomato seedlings across seasons for evaluating the impact of agrobacterial T6SS on disease occurrence and crown gall microbiota (termed gallobiome). Moreover, to overcome the challenge of interference of host organelles in the study of plant-associated microbiota, we optimized a blocker-based method for enriching true bacterial reads in 16S rRNA gene amplicon sequencing. Based on results from our infection assays, gallobiome composition analysis, as well as *in vitro* competition and colonization assays, this work indicates that the agrobacterial T6SS may provide competitive advantages on the plant surface for effective infection, leading to a higher disease incidence.

## RESULTS

### Environmental factors and the T6SS affect crown gall disease incidence.

**FIG 1:**
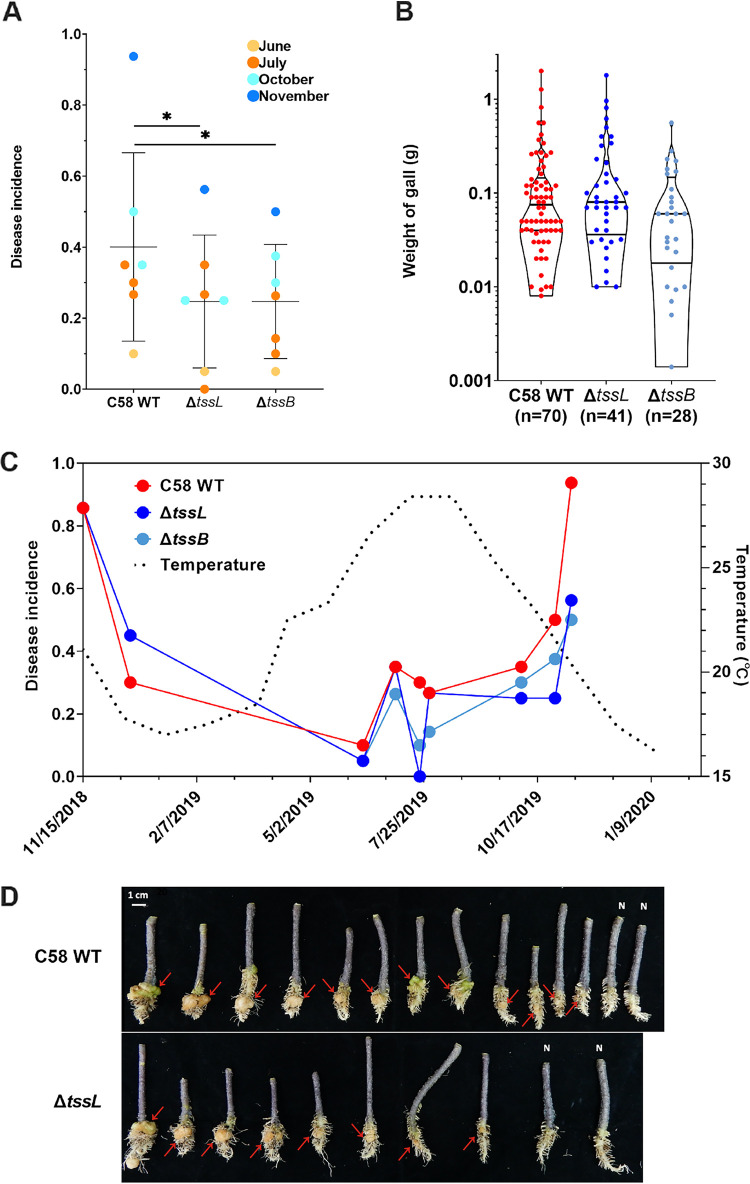
Tumorigenesis assay of agrobacterial strain C58 and its T6SS-deficienct mutants. For each experiment, 14 to 20 wounded tomato seedlings were grown in soil containing one of the strains tested (i.e., C58 WT, Δ*tssL*, and Δ*tssB*) and collected at 60 days postinoculation (dpi). (A) Disease incidence plotted by strain and color-coded according to the month of inoculation (light yellow, June; orange, July; light blue, October; dark blue, November). Lines and error bars indicate the mean ± SD. Statistical significance was tested using two-way ANOVA followed by Tukey’s multiple comparisons; *P* = 0.025 and 0.027 when comparing the wild type to Δ*tssL* and Δ*tssB*, respectively. (B) Weight distribution of the galls collected (see details in Table S5). No significant difference among the three strains (*P* = 0.15, Kruskal-Wallis test). (C) Correlation plot of disease incidence and temperature of daily average. (D) Crown galls generated through soil inoculation; example from inoculation on 15 November 2018. N, no gall formation in some of inoculated plants.

The wild-type (WT) strain C58 and two C58-derived T6SS mutants with deletion of essential T6SS genes, Δ*tssL* and Δ*tssB*, were used to study the effects of the agrobacterial T6SS in tumorigenesis using a soil inoculation method. Nine batches of inoculation experiments across different seasons were conducted in this study (Table 1). In total, 139 crown galls were collected, including 70 induced by the WT, 41 induced by Δ*tssL*, and 28 induced by Δ*tssB*. The results showed that all three strains are capable of inducing tumors, but the disease incidences of Δ*tssL* and Δ*tssB* were significantly lower than that of WT (Table 1, Fig. 1A). There was no significant weight difference among the crown galls induced by different strains (Fig. 1B). It is notable that there is an inverse correlation between disease incidence and temperature across seasons for all three strains throughout the year (Fig. 1C and D). These results indicated that the presence of a functional T6SS and the month of inoculation both affected the disease incidence under soil inoculation conditions.

**TABLE 1:** Incidences of crown gall disease on wounded tomato stems by soil inoculation

Batch	Date of inoculation (yr/mo/day)	Disease incidence (diseased/total inoculated seedlings)^*a*^		
		C58 WT	Δ*tssL*	Δ*tssB*
1	2018/11/15	0.86 (12/14)	0.86 (12/14)	ND^*b*^
2	2018/12/20	0.30 (6/20)	0.45 (9/20)	ND
3	2019/06/10	0.10 (2/20)	0.05 (1/20)	0.05 (1/20)
4	2019/07/04	0.35 (7/20)	0.35 (7/20)	0.26 (5/19)
5	2019/07/22	0.30 (6/20)	0.00 (0/20)	0.10 (2/20)
6	2019/07/29	0.27 (4/15)	0.27 (4/15)	0.14 (2/14)
7	2019/10/05	0.35 (7/20)	0.25 (5/20)	0.30 (6/20)
8	2019/10/30	0.50 (8/16)	0.25 (4/16)	0.38 (6/16)
9	2020/11/11	0.94 (15/16)	0.56 (9/16)	0.50 (8/16)

a Two-way ANOVA followed by Tukey’s multiple comparisons test on disease incidences of different strains indicated that the disease incidence of Δ*tssL* and Δ*tssB* was significantly lower than that of *Agrobacterium* C58 WT (*P* = 0.0225 and 0.0227, respectively).

b ND, not determined.

10.1128/mbio.00177-23.9 TABLE S5 Metadata of harvested crown galls. Download Table S5, DOCX file, 0.03 MB. Copyright © 2023 Wang et al. 2023 Wang et al. https://creativecommons.org/licenses/by/4.0/ This content is distributed under the terms of the Creative Commons Attribution 4.0 International license.

### Round I of 16S rRNA gene amplicon sequencing: initial trial.

To determine the gallobiome composition, crown galls with similar weights across seasons were selected for 16S rRNA gene amplicon sequencing. For round I, six crown gall DNA samples, three induced by the WT and three by Δ*tssL*, were amplified with two commonly used 16S rRNA gene primer sets, V3 to V4 and V5 to V7 (see Table S1A in the supplemental material). The Illumina sequencing produced 140,830 and 601,299 reads from the V3 to V4 and V5 to V7 sets, respectively. However, the majority (99.1% in V3 to V4 and 93.6% for V5 to V7) of these reads were derived from plant chloroplast and mitochondria. After removing these host contaminants, only 1,230 and 38,320 bacterial reads remained (Table 2).

**TABLE 2:** Sum of read counts in six tumor samples before and after filtering nonbacterial reads from amplicon sequencing round I for protocol test

16S rRNA regions	No. of reads		Percentage of nonbacterial reads (%)
	Before filtering	After filtering	
V3–V4	140,830	1,230	99.1
V5–V7	601,299	38,320	93.6

10.1128/mbio.00177-23.5 TABLE S1 **A.** 16S rRNA gene primer sets used in this study **B.** Corresponding blockers (3′ modified oligonucleotides with C_3_ spacer). Download Table S1, DOCX file, 0.02 MB. Copyright © 2023 Wang et al. 2023 Wang et al. https://creativecommons.org/licenses/by/4.0/ This content is distributed under the terms of the Creative Commons Attribution 4.0 International license.

### Round II of 16S rRNA gene amplicon sequencing: optimization.

Due to the severe host contamination observed in round I, that used the universal 16S rRNA gene primers, we evaluated the performance of different 16S rRNA gene primers with blockers (3′ modified oligonucleotides with C3 spacer) that could prevent the amplification of tomato chloroplast and mitochondrial rRNA genes (22–24).

**FIG 2:**
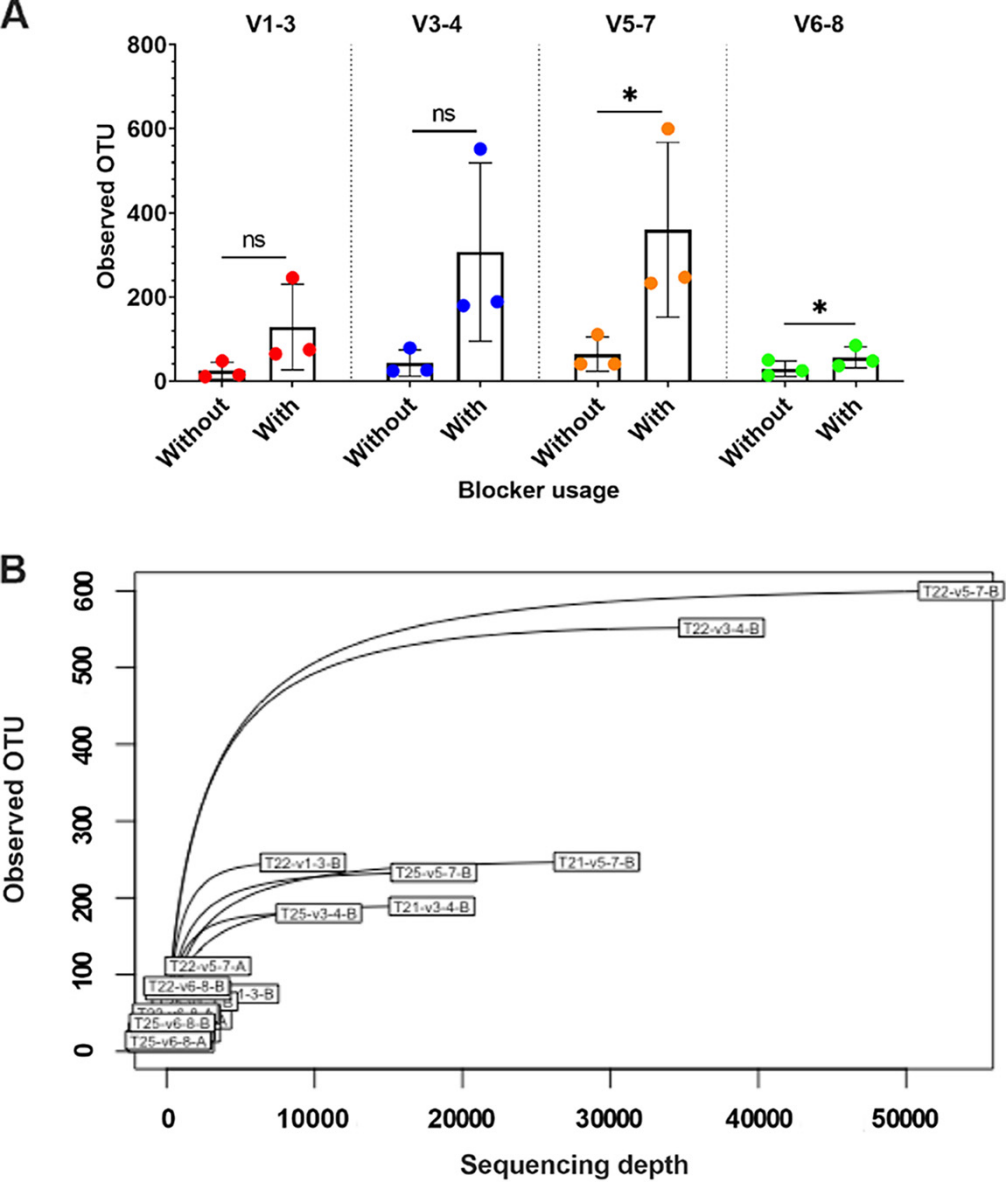
Optimizing 16S rRNA gene amplification by using PCR blockers. Three crown gall samples (named 21W, 22W, and 25W) induced by the C58 wild type were used for analysis. V1 to V3, V3 to V4, V5 to V7, and V6 to V8 represent different primer pairs targeting different variable regions on the 16S rRNA gene. (A) The OTU counts of 16 rRNA gene amplicons after filtering nonbacterial OTUs. (ns, not statistically significant; *, *P *< 0.05, Student’s *t* test). The bar indicates the mean ± SD of OTUs. (B) Alpha rarefaction curves of the observed bacterial OTUs based on data sets from amplicon sequencing round II. Sample size on the *x* axis indicates different subsampling depths of each data set.

**FIG 3:**
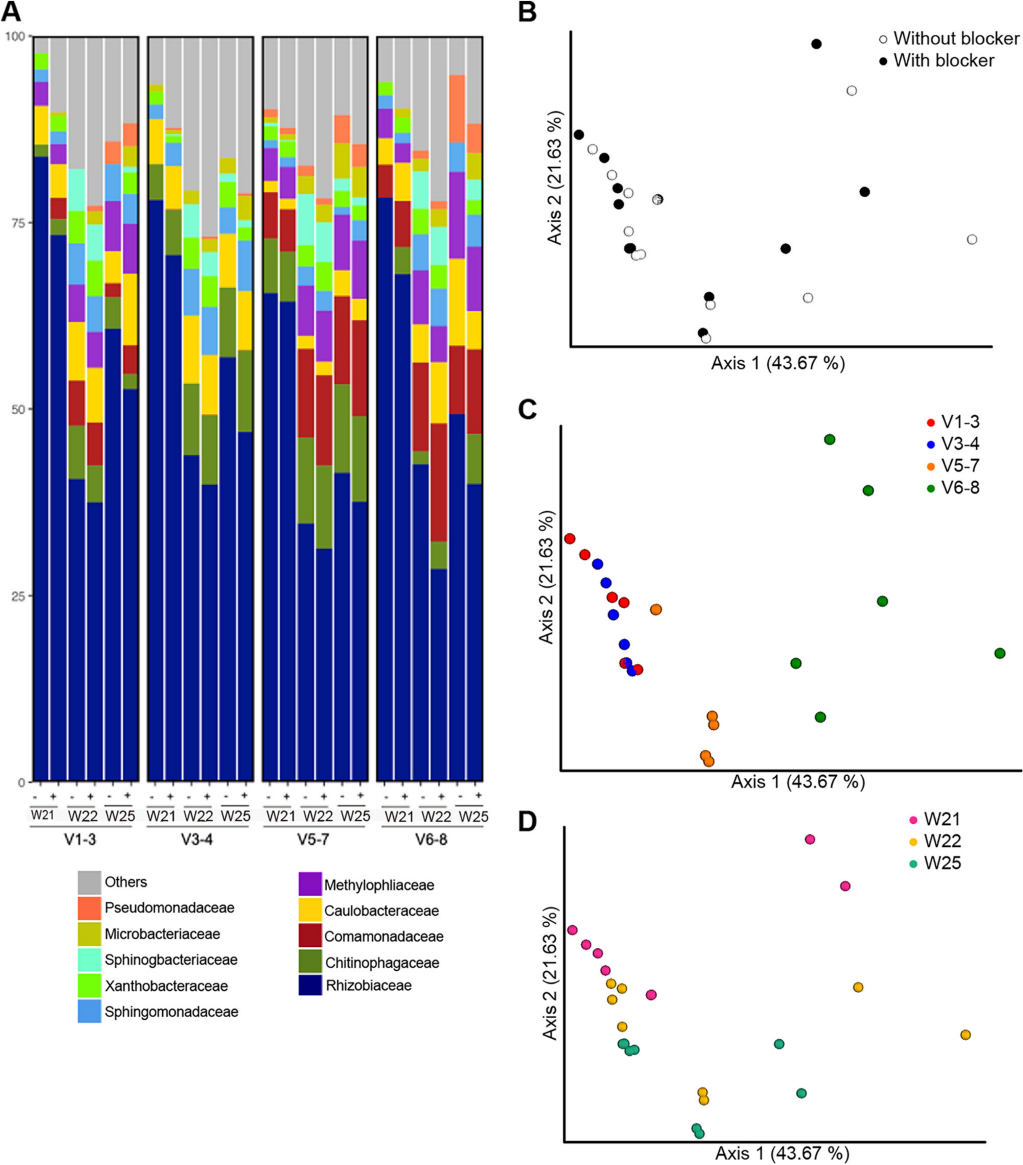
Gallobiome composition when using different primers amplified with or without blockers. (A) Different primer sets (V1 to V3, V3 to V4, V5 to V7, and V6 to V8) for analysis of three crown gall samples (21W, 22W, and 25W; see details in Table S6) induced by the C58 wild type are indicated. The top 10 observed families are listed. –, amplification without blockers; +, amplification with blockers. (B to D) Principle-coordinate analysis (PCoA) plots of the bacterial composition of the data sets were generated based on the weighted UniFrac matrix, and color coded by (B) blocker usage (*P* = 0.068, *R*^2^ = 0.026, ADONIS), (C) different primer sets (*P* = 0.001, *R*^2^ = 0.524, ADONIS), and (D) different crown gall samples (*P* = 0.001, *R*^2^ = 0.238, ADONIS).

Based on the alignment of bacterial, tomato chloroplast, and mitochondrial 16S rRNA gene sequences (Fig. S1), we designed the primer sets and cognate blockers against different variable regions in 16S rRNA genes (Table S1A and B). The new primer sets and blockers were tested using the three WT-induced crown gall DNA samples from round I. After sequencing and data processing, the result indicated that the blockers have variable effectiveness in reducing host contamination (Table 3, Fig. 2A). For the V3 to V4 and V5 to V7 primer sets, adding blockers increased the bacterial reads to 5.3 to 41.5% of total reads, compared to only 0.2 to 1.9% of bacterial reads per sample without blockers. For the V1 to V3 and V6 to V8 primer sets, adding blockers increased the bacterial reads to only 1.6 to 5.6% of total reads per sample. The reads were grouped into species-level operational taxonomic units (OTUs) with 99% sequence identity. The alpha rarefaction curves based on the OTU counts indicated that, for those crown gall samples, the curves would approach saturation when the sequencing depth was over about 10,000 reads per sample (Fig. 2B). According to the bar plot of bacterial composition at the family level, we observed a higher number of families identified from the same crown galls amplified by adding blockers (Fig. 3A). The result of principal-coordinate analysis (PCoA) indicated that adding blockers did not cause significant biases in the inferred microbiota composition (Fig. 3B). However, the use of different primer sets resulted in a significant difference in the inferred microbiota composition (Fig. 3B and C), which has been reported previously (25).

**TABLE 3:** Read count before and after filtering nonbacterial ASVs from the three crown gall DNA samples (named W21, W22, and W25) induced by WT *Agrobacterium* C58

Sample ID	Dataset ID	No. of reads before filtering	No. of reads after filtering chloroplast and mitochondrial DNA	Percentage of nonhost reads (%)
1115-21W	T21-v1-3-B	176,544	9,914	5.6
1115-22W	T22-v1-3-B	183,475	5,608	3.1
1115-25W	T25-v1-3-B	152,387	2,404	1.6
1115-21W	T21-v1-3-A	185,107	853	0.5
1115-22W	T22-v1-3-A	247,240	379	0.2
1115-25W	T25-v1-3-A	178,980	220	0.1
1115-21W	T21-v3-4-B	239,243	38,462	16.1
1115-22W	T22-v3-4-B	246,063	19,218	7.8
1115-25W	T25-v3-4-B	209,991	11,185	5.3
1115-21W	T21-v3-4-A	200,185	979	0.5
1115-22W	T22-v3-4-A	205,966	796	0.4
1115-25W	T25-v3-4-A	193,350	426	0.2
1115-21W	T21-v5-7-B	129,422	53,715	41.5
1115-22W	T22-v5-7-B	140,012	29,078	20.8
1115-25W	T25-v5-7-B	135,692	18,052	13.3
1115-21W	T21-v5-7-A	150,015	2,792	1.9
1115-22W	T22-v5-7-A	139,974	1,507	1.1
1115-25W	T25-v5-7-A	133,035	786	0.6
1115-21W	T21-v6-8-B	92,167	3,691	4.0
1115-22W	T22-v6-8-B	92,053	3,197	3.5
1115-25W	T25-v6-8-B	82,361	2,077	2.5
1115-21W	T21-v6-8-A	121,145	1,604	1.3
1115-22W	T22-v6-8-A	138,186	1,273	0.9
1115-25W	T25-v6-8-A	121,820	708	0.6
	NC-v3-4-B	1,012	979	96.7

10.1128/mbio.00177-23.1 FIG S1 DNA sequence alignment of 16S rRNA genes of selected bacterial strains and tomato Known-You 301 chloroplast and mitochondria. The 16S rRNA gene sequences of chloroplasts and mitochondria in *Solanum lycopersicum* (tomato) cultivar Known-You 301 were obtained from this study. All of the bacterial 16S rRNA gene sequences were accessed from the Reference Sequence (RefSeq) database of the National Center of Biotechnology Information (NCBI). The species, strain name, and accession number of the 16S rRNA gene are listed to the left of each sequence. The sequences mentioned above were aligned in MEGAX via ClustalW multiple alignment with a gap opening penalty of 15.00 and a gap extension penalty of 6.66 (default). The levels of shaded blue reflect the degree of identity. The regions of each primer and blocker are underlined. The sequences highlighted by red, black, yellow, and green are the annealing region of primer sets for V1 to V3, V3 to V4, V5 to V7, and V6 to V8 in the 16S rRNA gene, respectively. The gray framed sequences in tomato chloroplast and mitochondrial 16S rRNA genes are the blockers for cognate primers. Download FIG S1, PDF file, 1.5 MB. Copyright © 2023 Wang et al. 2023 Wang et al. https://creativecommons.org/licenses/by/4.0/ This content is distributed under the terms of the Creative Commons Attribution 4.0 International license.

10.1128/mbio.00177-23.10 TABLE S6 Crown gall metadata used for analysis of amplicon sequencing round III. Download Table S6, DOCX file, 0.02 MB. Copyright © 2023 Wang et al. 2023 Wang et al. https://creativecommons.org/licenses/by/4.0/ This content is distributed under the terms of the Creative Commons Attribution 4.0 International license.

Overall, adding blockers at the PCR step increased bacterial read counts and the resolution of the gallobiome. Because the use of V5 to V7 primers and cognate blockers obtained the highest number and percentage of bacterial reads, the V5 to V7 setup with blockers was selected for 16 rRNA gene amplicon sequencing of crown galls induced by WT and T6SS mutants.

### Round III of 16S rRNA gene amplicon sequencing: impact of the T6SS on the gallobiome.

**FIG 4:**
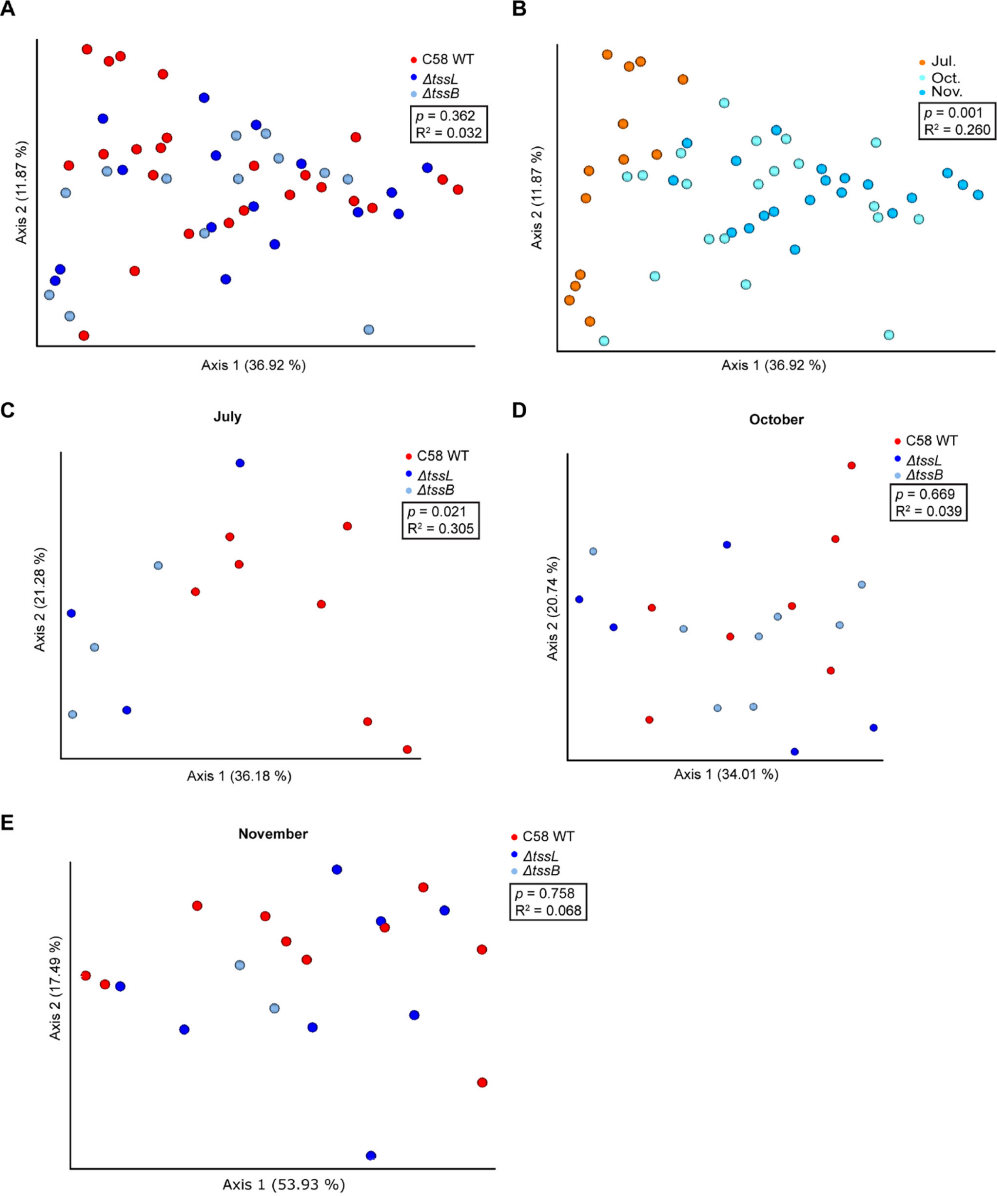
Principle-coordinate analysis (PCoA) of bacterial composition in 53 crown galls induced by the C58 WT and T6SS mutants. The plots were drawn based on the weighted UniFrac matrix of the bacterial communities. (A and B) Crown galls induced by different strains (C58 WT, Δ*tssB*, Δ*tssL*) (A) or inoculated in different months (B) are labeled with different colors in each panel. (C to E) The data sets were further split based on the month of inoculation, and the PCoA plots of gallobiomes from (C) July, (D) October, and (E) November are shown. Statistical differences in clustering were evaluated via ADONIS permutation test, and the corresponding *P* and *R*^2^ values are indicated.

Among all of the 139 crown galls collected, 53 tumors in the range of 0.06 to 0.56 g (24 induced by WT, 16 by Δ*tssL*, and 13 by Δ*tssB*) were used for the analysis. We obtained an average of 69,980 ± 26,452 (mean ± standard deviation [SD]) reads per sample, ranging from 9,814 to 116,072 reads per sample. Before the analysis, the amplicon sequence variants (ASVs) were clustered into species-level OTUs, and the singletons were removed. Based on the alpha rarefaction curves and the minimal read counts of the samples (Fig. S2), diversity analysis was conducted with data set subsampling at 9,800 reads per sample. The result of PCoA showed that the gallobiomes induced by the WT and two T6SS mutant strains were not significantly different (Fig. 4A). Most of the variation between samples was contributed by different seasons (July versus October/November) (Fig. 4B). After splitting and reanalyzing the data set based on the month of inoculation, we observed the difference between the gallobiomes associated with the WT and two T6SS mutants in July (Fig. 4C). In contrast, no difference was detected in those galls induced in October or November (Fig. 4D and E).

10.1128/mbio.00177-23.2 FIG S2 Alpha rarefaction curves of the observed bacterial OTUs based on amplicon sequencing of 53 gall samples in sequencing round III. The rarefaction curves were plotted after clustering DADA2-output ASVs into 99% OTUs. Sample size in the *x* axis indicates different subsampling depths of each dataset; the *y* axis shows the number of observed OTUs under certain subsampling depths. The label of each curve indicates the sample ID in metadata. Download FIG S2, PDF file, 0.7 MB. Copyright © 2023 Wang et al. 2023 Wang et al. https://creativecommons.org/licenses/by/4.0/ This content is distributed under the terms of the Creative Commons Attribution 4.0 International license.

**FIG 5:**
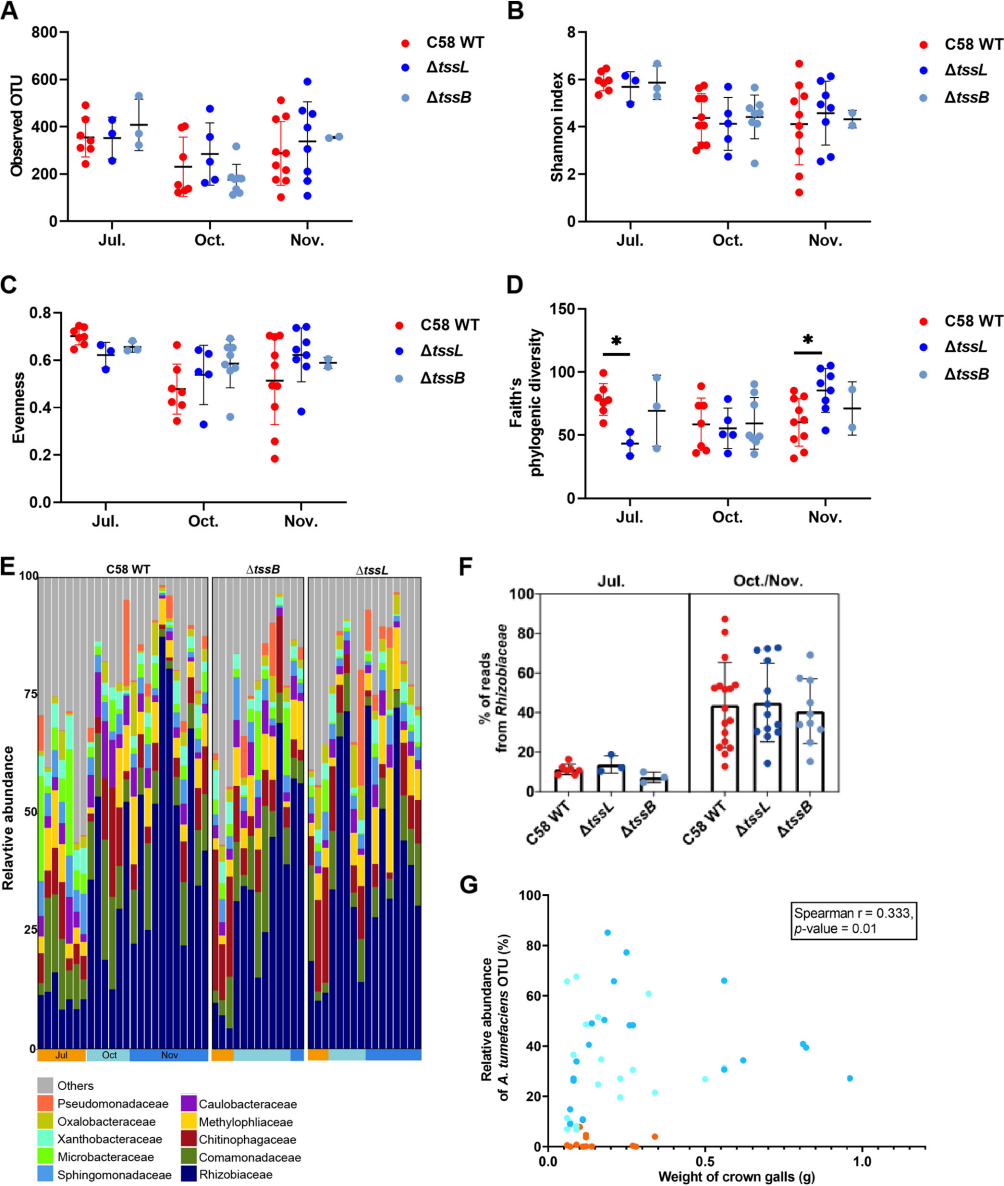
Alpha diversity and composition of crown galls generated in different months by the C58 WT and T6SS mutants. (A to D) The (A) Observed OTU, (B) Shannon index, (C) evenness, and (D) Faith’s phylogenic diversity of each sample generated in different months are indicated. Lines and error bars indicate the mean ± SD. Two-way ANOVA followed by Tukey’s multiple-comparison test were performed, and the asterisks indicate the statistically significant difference between two strains (*P* < 0.05). (E) Bacterial composition of 53 crown galls. For each strain, the samples are grouped by the month of inoculation. The top 10 most abundant families are listed and colored differently, and the remaining ones are combined into “others.” (F) Relative abundance of *Rhizobiaceae* in galls inoculated in different months and strains. (G) Scatterplot of the relative abundance of agrobacteria OTU in gallobiome—weight of the crown galls. A positive correlation between the weight of crown galls and the relative abundance of *Agrobacterium* C58 was observed (Spearman *r* = 0.333, *P* = 0.01).

The alpha diversity indices, including observed OTUs, Shannon index, and Pielou’s evenness index had no significant difference between the gallobiomes associated with the WT and T6SS mutants (Fig. 5A to C). Interestingly, we found that in galls induced in July, the WT gallobiome showed significantly higher Faith’s phylogenic diversity than the gallobiome associated with Δ*tssL*; and in galls induced in November, the Δ*tssL* gallobiome showed higher diversity than the WT gallobiome (Fig. 5D). The gallobiome associated with Δ*tssB* also exhibited higher diversity than the WT gallobiome, but the difference was not statistically significant.

While there were variations in bacterial compositions among crown galls, the top 10 bacterial families in gallobiomes were quite consistent: *Rhizobiaceae*, *Comamonadaceae*, *Chitinophagaceae*, *Methylophilaceae*, *Caulobacteraceae*, *Sphingomonadaceae*, *Microbacteriaceae*, *Xanthobacteraceae*, *Oxalobacteraceae*, and *Pseudomonadaceae* (Fig. 5E). The bacterial family *Rhizobiaceae*, which *Agrobacterium* belongs to, was found to be highly variable and accounted for 5 to 85% of the entire community. The most abundant OTU in this *Rhizobiaceae* data set was 100% matched to 16S rRNA genes of C58, suggesting that the abundance of this OTU could be referred to the relative abundance of WT or mutant inoculum in crown galls. When the weight of crown galls was plotted against the relative abundance of this OTU in gallobiomes, we found that the abundance of *Rhizobiaceae* in July was dramatically lower than those in October and November (Fig. 5F), but there was no significant difference observed among different strains. Furthermore, a positive correlation between the weight of crown galls and the relative abundance of this agrobacterial OTU was observed (Fig. 5G).

### *Sphingomonadaceae* and *Burkholderiaceae* were more abundant in the gallobiomes induced by the T6SS mutants in July.

**FIG 6:**
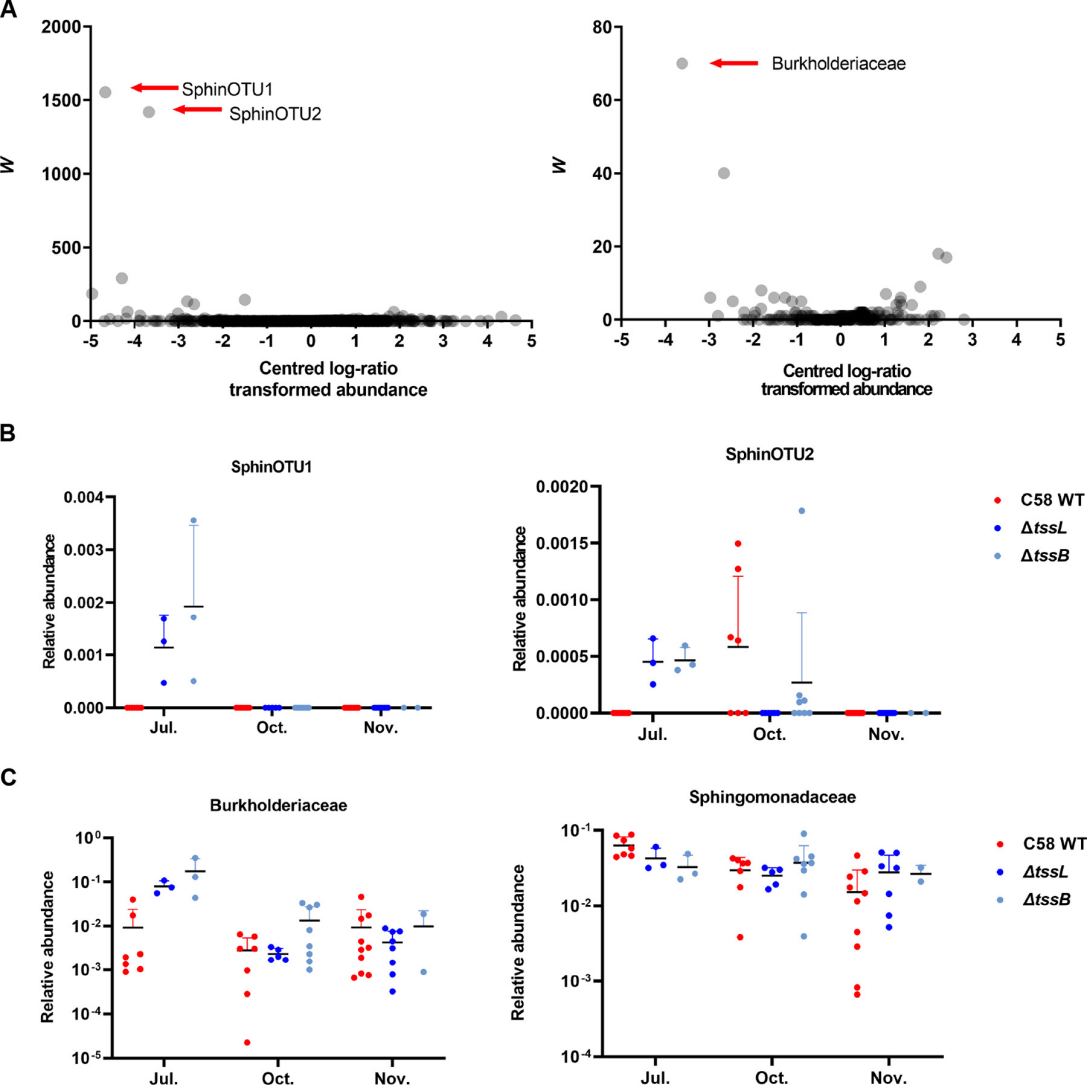
Analysis of the composition of microbiomes (ANCOM) for gallobiomes in July. (A) The ANCOM volcano plots were drawn based on the centered-log-ratio-transformed abundance of OTUs or bacterial families. The taxa that exhibit differential abundance (DA) between gallobiomes induced by the C58 WT and T6SS mutants are indicated by red arrows. The two DA OTUs belonging to *Sphingomonadaceae* were named SphinOTU1 and SphinOTU2. (B and C) The relative abundance of focal taxa. Lines and error bars indicate the mean ± SD.

Next, we analyzed the species-level OTUs and families with differential abundance (DA) between gallobiomes associated with the WT and T6SS mutants in July. Two OTUs belonged to *Sphingomonadaceae*, here named SphinOTU1 and SphinOTU2, and *Burkholderiaceae* family members were significantly enriched in the gallobiomes induced by T6SS mutants (Fig. 6A). SphinOTU1 was present in gallobiomes only in July but not in October/November, and SpinOTU2 was present in both July and October but not in November (Fig. 6B). Neither SpinOTU1 nor SpinOTU2 was identified in gallobiomes induced by the WT in July. *Burkholderiaceae* was enriched in gallobiomes induced by the T6SS mutants in July, but there was no consistent enrichment in October and November (Fig. 6C). Although SphinOTU1 and SphinOTU2 were only present in gallobiomes induced by T6SS mutants in July, at the family level, *Sphingomonadaceae* was not enriched in a T6SS-dependent manner in any months (Fig. 6C).

### T6SS-dependent antagonism between agrobacteria and a *Sphingomonas* sp. isolate.

**FIG 7:**
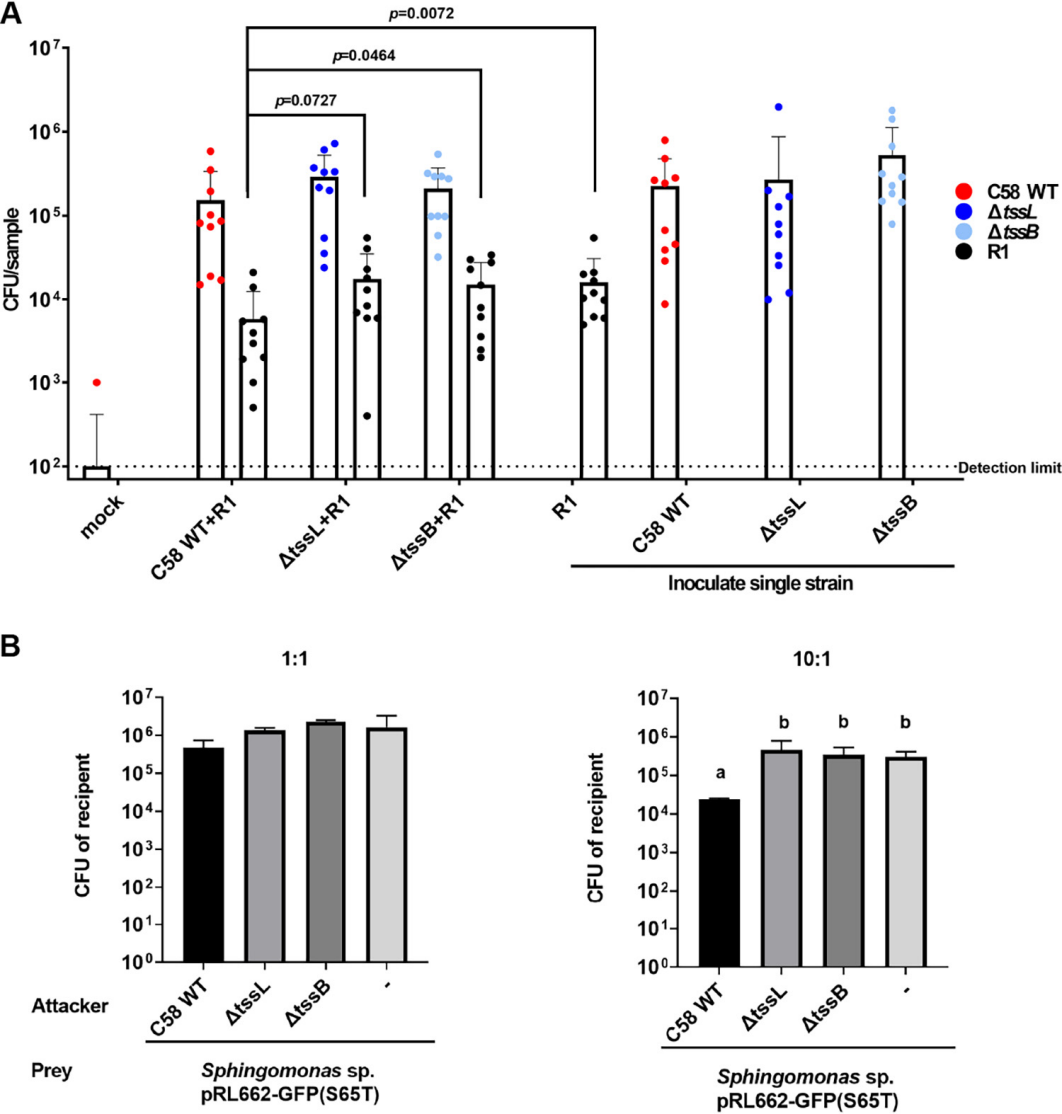
Competition between agrobacteria and *Sphingomonas* sp. R1 *in vitro* and on wound sites of tomato stems. Each of the three *Agrobacterium* strains (i.e., C58 WT, Δ*tssL*, and Δ*tssB*) was individually tested for competition against *Sphingomonas* sp. R1 isolated from rhizosphere of tomato. (A) *Agrobacterium* only or coinfected with *Sphingomonas* sp. R1 at 1:1 ratio in soil was recovered from the surface of wounded stem segments at 10 dpi, and the CFU of *Agrobacterium* and *Sphingomonas* sp. R1 were plotted. A *t* test indicated the CFU differences of *Sphingomonas* sp. R1 inoculation alone or coinfected with T6SS mutants compared to WT with indicated *P* values. Data are the mean ± SD of three independent experiments, each with five seedlings inoculated for each strain. The ANOVA test indicated no significant difference among means (*P* *=* 0.0945 and 0.0551 for *Agrobacterium* and *Sphingomonas* sp. R1 CFU, respectively). (B) The *in vitro* competition results based on *Agrobacterium*: *Sphingomonas* sp. R1 at 1:1 and 10:1 ratios and the survival of *Sphingomonas* sp. R1 were plotted; lines and error bars indicate the mean ± SD of four biological replicates from two independent experiments. Based on ANOVA followed by Tukey’s multiple-comparison test, the C58 WT significantly reduced *Sphingomonas* sp. R1 CFU when mixed in a 10:1 ratio (*P* = 0.0153) but not in a 1:1 ratio (*P* = 0.2753).

The T6SS-dependent differential abundance of two *Sphingomonas* OTUs in gallobiomes motivated us to investigate whether C58 exhibits T6SS antibacterial activity to *Sphingomonas.* A *Sphingomonas* sp. strain, R1, isolated from the tomato rhizosphere in one soil inoculation experiment was used for interbacterial competition assays *in planta* and *in vitro*. Each of the agrobacterial strains was mixed at a 1:1 ratio with *Sphingomonas* sp. R1 as inoculum, and soil inoculation on wounded tomato seedlings was performed. The colonization efficiency of those strains was determined by counting the CFU recovered from wounded stem segments at 10 days postinoculation (dpi). The results showed an ~0.5-log reduced CFU of *Sphingomonas* sp. R1 when it was coinoculated with the WT compared to that with Δ*tssL* and Δ*tssB* or R1 only (Fig. 7A). The recovered CFU of agrobacterial WT and T6SS mutants inoculated alone or with *Sphingomonas* sp. R1 were 1 to 1.5 log higher than that of *Sphingomonas* sp. R1, but there was no difference among agrobacterial WT and T6SS mutants inoculated alone or with *Sphingomonas* sp. R1. These results suggest that agrobacteria have higher competitive colonization efficiency than *Sphingomonas* sp. R1, in part dependent on a functional T6SS. Accordingly, an interbacterial competition assay on agar plates also showed T6SS-dependent antibacterial activity to *Sphingomonas* sp. R1 (Fig. 7B). However, when interbacterial competition was carried out *in vitro* on agar plates, only weak antibacterial activity was observed at 1:1 ratio, but ~0.5-log reduced CFU of *Sphingomonas* sp. R1 was detected when competition was carried out at a 10:1 ratio of agrobacteria to *Sphingomonas* sp. R1. The results show that the T6SS provides agrobacteria with a higher competitive advantage against *Sphingomonas* sp. R1 on plant wounding sites than *in vitro.* We further evaluated whether the number of CFU of *Sphingomonas* sp. R1 was enriched in crown galls induced by T6SS mutants compared to those induced by the WT via the soil inoculation method used for 16S rRNA gene amplicon sequencing. Surprisingly, *Sphingomonas* sp. R1 was not always present in crown galls at 28 dpi, and R1 was recovered from only one out of three independent experiments by direct induction of crown galls on tomato stem (Fig. S3). The abundance of C58 ranged from 10^4^ to 10^7^ CFU per gall, and no consistent difference could be observed between the WT and the T6SS mutants. Together, these results suggest that C58 exhibits antibacterial activity to inhibit the *in vitro* growth and plant colonization of *Sphingomonas* sp. R1. The T6SS may help agrobacteria to gain competitive growth over other competing rhizobacteria on the plant surface for effective infection, leading to higher disease incidence.

10.1128/mbio.00177-23.3 FIG S3 Competition of *Agrobacterium* and *Sphingomonas* sp. R1 in tomato gall on stems at 28 dpi. Each of the three *Agrobacterium* strains (i.e., C58 WT, Δ*tssL*, and Δ*tssB*) and R1 were mixed at a 1:1 ratio and then inoculated on wounded tomato stems. The galls were harvested and homogenized for plating at 28 dpi. *Agrobacterium* strains and *Sphingomonas* sp. R1 were recovered on 523 medium plates containing the proper antibiotics. CFU data for agrobacteria are the mean ± SD of three independent experiments/batches, each with five seedlings inoculated for each strain. R1 could only be recovered from galls in batch 3. The *P* values of ANOVA against CFU numbers of C58 for each batch are 0.786, 0.0023, and 0.230; the *P* value of ANOVA against CFU numbers of R1 is 0.6413. The significance of CFU numbers of C58 in the second batch is due to the low CFU counts of the Δ*tssL* single-inoculation group, which could not be replicated in other two batches. Download FIG S3, PDF file, 0.3 MB. Copyright © 2023 Wang et al. 2023 Wang et al. https://creativecommons.org/licenses/by/4.0/ This content is distributed under the terms of the Creative Commons Attribution 4.0 International license.

## DISCUSSION

In this study, we designed experiments to address whether the T6SS affects agrobacterial tumorigenesis and gallobiome composition. By establishing a soil inoculation platform to evaluate the disease incidence of wounded tomato seedlings across seasons, we revealed that a functional T6SS is positively correlated with disease occurrence but not gall weight. Furthermore, by developing an effective protocol for enrichment of bacterial 16S rRNA genes by utilizing blocker to prevent host contamination, we found that seasons or environmental factors are the major drivers in shaping gallobiome composition, while the T6SS could also influence microbiota in a more specific manner.

The evidence that the agrobacterial T6SS promotes tumorigenesis in soil inoculation (Table 1, Fig. 1) but not direct inoculation (19) suggests that the T6SS is not directly involved in virulence. Instead, agrobacteria may deploy the antibacterial weapon to increase the occurrence of infection in the presence of relatively complex microbial communities in rhizosphere (Table S2) (26). Once galls are induced, the T6SS is not required for gall development since no significant difference in weight was observed between the WT and T6SS mutants (Fig. 1B). The presence of a functional T6SS has no impact on the population size of agrobacteria, which is consistent with recent findings that T6SS genes were not identified as fitness genes by a transposon insertion sequencing (Tn-seq) screen in crown galls (27). The T6SS of C58 also appears not to be the key factor in shaping gallobiome composition, suggesting that agrobacteria may also use other methods of niche competition in galls. It is generally believed that the agrobacteria inciting crown galls and later becoming residents have privilege to access the specific opines synthesized by transformed plant cells containing the agrobacterial T-DNA (28). This opine concept has not been experimentally validated but is supported by a study showing that the ability of agrobacteria to trap opines can be a competitive advantage over siblings which cannot utilize opines (29). Therefore, it would be interesting to investigate whether opine synthesis and other fitness genes would impact the crown gall microbiota in which the T6SS may synergistically enhance the niche occupation.

10.1128/mbio.00177-23.6 TABLE S2 Identification of bacterial isolates from tomato rhizosphere. Download Table S2, DOCX file, 0.01 MB. Copyright © 2023 Wang et al. 2023 Wang et al. https://creativecommons.org/licenses/by/4.0/ This content is distributed under the terms of the Creative Commons Attribution 4.0 International license.

The findings that disease incidence and crown gall weight are inversely correlated with temperature (Fig. 1C) are expected. Early studies demonstrated that crown gall sizes were dramatically decreased when the host plants were inoculated at 28 to 30°C, while there was no gall formation at 31°C or above (30, 31). In addition, the T4SS complex and its associated T-pilus are more stable or produced at higher levels at 19°C than 28°C, whereas T4SS-mediated plasmid conjugation is deficient at 28°C (32–36). The increased tumor weight when inoculation occurred in fall/winter is also correlated with a higher relative abundance of agrobacterial OTU (Fig. 5G), suggesting the active proliferation of the agrobacterial population inside crown galls. Furthermore, our data show that crown galls produced in summer have distinct microbiota compared to those from fall/winter (Fig. 4B), which echoes previous studies of Allorhizobium vitis showing that the crown gall microbiota of grapevine is significantly different in summer (37). Interestingly, while there is no global difference in the gallobiome induced by the WT or T6SS mutants inoculated in fall/winter, we found that the two *Sphingomonadaceae* OTUs and the family *Burkholderiaceae* were only present or significantly enriched in the gallobiome induced by T6SS mutants in summer (Fig. 6). By counting viable agrobacterial inoculum, we showed that C58 exhibits a T6SS-mediated antibacterial activity to *Sphingomonas* sp. R1 *in vitro* and in rhizosphere (Fig. 7A and B), but there was no significant difference in R1 abundance between the galls coinfected with C58 WT or T6SS mutants (Fig. S3). Interestingly, we noticed that the colonization efficiency of those T6SS mutants was more variable compared to that of those of the WT from the combined data set (Fig. S4). To this end, it remains to be determined whether the absence or reduced abundance of two *Sphingomonadaceae* OTUs and *Burkholderiaceae* in gallobiome induced by the WT compared to that induced by T6SS mutants is a direct consequence of antibacterial activity conferred by the T6SS. However, current evidence suggests that agrobacteria may use the T6SS to gain a competitive advantage by its antibacterial activity against other bacterial species residing in the soil or rhizosphere and therefore increase its success in inciting tumors on infected plants.

10.1128/mbio.00177-23.4 FIG S4 Agrobacterial colonization on wounded stem segments. The colonies of *Agrobacterium* strains C58 WT, Δ*tssL*, and Δ*tssB* recovered from the surface of wounded stem segments following the soil inoculation procedure were plotted. Different symbols indicate the outcome from different batches of the colonization assay. The *F* test indicates that the variance of Δ*tssL* and Δ*tssB* was significantly different compared to C58 WT (*p*[*F* ≤ *f*] = 0.00028 and 1.77826E-05, respectively). Brown-Forsythe and Welch ANOVA tests indicated no significant difference among means (*P* = 0.4847). The line indicates the median. Download FIG S4, PDF file, 0.2 MB. Copyright © 2023 Wang et al. 2023 Wang et al. https://creativecommons.org/licenses/by/4.0/ This content is distributed under the terms of the Creative Commons Attribution 4.0 International license.

Besides the *Rhizobiaceae*, which agrobacteria belong to, other bacterial families also occupied considerable proportions of crown gall microbiota, such as *Comamonadaceae*, members of which are commonly found in soil and water, as well as the plant-associated bacterium *Variovorax*, shown to be important for root growth of *Arabidopsis* (38, 39). Bacteria belong to the *Xanthobacteraceae* and *Pseudomonadaceae*, the two families highly associated with plants as either pathogens or commensals, also had high relative abundance in the crown galls. Some Pseudomonas species, such as P. fluorescens and P. putida are capable of utilizing opines (40, 41), which may explain why bacterial species other than the agrobacterial inoculum could also colonize and propagate in high relative abundance in crown galls.

We found that the predominance of plant DNA in crown gall samples led to the low resolution of the endophytic bacterial microbiota via standard 16S rRNA gene amplicon sequencing due to the coamplification of chloroplast and mitochondrial sequences. Although the use of 16S rRNA gene primers containing mismatches to the 16S rRNA gene of plant chloroplast or mitochondria could potentially reduce host contamination in endophytic microbiota studies (25, 26), the bacteria-specific primers resulted in >90% of host reads of our initial attempt (round I) (Table 2), which is similar to results of a previous study (22). When adding blockers that could specifically anneal to chloroplast or mitochondrial DNA but not be extended by DNA polymerase during PCR, we successfully reduced the host contamination and obtained sufficient sequencing depth for this study. Our results support the effectiveness of blockers in increasing bacterial reads of the endophyte community (22–24). Our newly developed soil inoculation method coupled with blocker-mediated enrichment of bacterial 16S rRNA gene amplicon sequencing (SI-BBacSeq) protocol for gallobiomes may be applicable to microbiota associated with tomato or even other plants after modifying the blocker sequences.

To date, collective plant microbiota studies suggest the enrichment of T6SS genes in microbiota as a trait for niche competition in microbiota (5, 8). However, the role and impact of the T6SS in different plant and microbiome contexts may be different. Our study suggests that the T6SS may provide agrobacteria with competitive advantages at the initial stage of infection but is likely not critical for proliferation once they established their niche inside the crown galls. Future studies to investigate the spatial distribution of agrobacterial cells and other endophytes inside crown galls or plant wounding sites, together with temporal and spatial visualization of the T6SS activity, are important for dissecting the roles of T6SS in agrobacterial disease ecology.

## MATERIALS AND METHODS

### Bacterial strains and growth conditions.

The bacterial strains and plasmids used in this study are listed in Table S3A. *Agrobacterium* strain C58 wild type and two T6SS mutants (i.e., Δ*tssB* and Δ*tssL*) (42) were first streaked on 523 medium agar plates (43) and incubated at 25°C for 48 h. *Sphingomonas* sp. R1 was grown on R2A medium without soluble starch and pyruvate (44). Escherichia coli was grown on lysogeny broth (LB) medium at 37°C. Freshly grown colonies were inoculated into corresponding broth for overnight culture. The concentrations and antibiotics used are 10 μg/mL gentamicin for E. coli, 50 μg/mL gentamicin for *Agrobacterium*, and 20 μg/mL kanamycin and 50 μg/mL gentamicin for *Sphingomonas* sp. R1.

10.1128/mbio.00177-23.7 TABLE S3 **A.** Bacterial strains and plasmids used in this study **B.** Primers used for plasmid construction. Download Table S3, DOCX file, 0.02 MB. Copyright © 2023 Wang et al. 2023 Wang et al. https://creativecommons.org/licenses/by/4.0/ This content is distributed under the terms of the Creative Commons Attribution 4.0 International license.

### Construction of strains enables antibiotic selection.

The primers used for construction are listed in Table S3B. The DNA fragment containing the gentamicin resistance (Gm^R^) gene and green fluorescence protein *gfp* (S65T) were amplified from pRL662::GFP(S65T) with the primer pair BclI-GFP-GmR-F-25 and BclI-GFP-GmR-R-25 and then cloned into XbaI-digested pJQ-COM. The newly constructed plasmid was named pJQ-com-GmR-GFP, and it enabled the generation of the Gm^R^ gene and *gfp* (S65T) knocked in upstream of *actC* (*atu5330*) of the *Agrobacterium* genome after double crossover. The knock-in strains were generated in an *Agrobacterium* C58, Δ*tssB*, and Δ*tssL* background. The knock-in event and excision of the pJQ200ks backbone were confirmed by green fluorescent protein (GFP) signal, gentamicin resistance, and PCR with the primer pair 3′sacB-5′GmR pJQ200 F and 3′sacB-5′GmR pJQ200 R, which target the fragment between *sacB* and the Gm^R^ gene on pJQ200ks. The plasmids pRL662::GFP(S65T) and pBBR::mCherry, conferring kanamycin resistance, were each transformed into *Sphingomonas* sp. R1 through electroporation. The procedure of electroporation and double crossover via pJQ200ks was performed as described previously (45).

### Plant material and growth conditions.

Seeds of Solanum lycopersicum (tomato) cultivar Known-You 301 from the Known-You Seed Co., Ltd. (Kaohsiung, Taiwan) were germinated and grown in unsterilized potting mixture (Jiffy premium fine peat substrate, perlite, and vermiculite, mixed in a 4:1:1 ratio [vol/vol/vol]) in greenhouse EL329 (N25.043047890856812, E121.61135464167913, greenhouse building, Academia Sinica, Taipei, Taiwan).

### Soil inoculation.

The soil inoculation method was optimized based on the method used for agrobacterial infection of pea and walnut (46, 47). Overnight (14 to 16 h) culture of the agrobacterial strain in 12 mL of 523 was were centrifuged at 6,000 × *g* for 10 min. The pellets were washed once using 10 mL of sterile saline (0.9% NaCl in H_2_O). The washed pellets were centrifuged and resuspended in sterile saline with the optical density at 600 nm (OD_600_) adjusted to 1. The bacterial suspension was mixed into unsterilized soil (1 mL suspension per 100 g of soil), which is expected to result in ~10^7^ CFU/g of soil, the optimized concentration that could incite crown galls but not at 100%. Tomato seedlings with two true leaves (2 to 3 weeks old) were wounded at the site between the primary root and cotyledons using a fire-sterilized sewing needle, planted in the inoculated soil, and grown in the greenhouse. Each strain was inoculated with 14 to 20 tomato seedlings, and the inoculation with saline was used as a negative control.

### Harvest, surface sterilization, and storage of crown gall samples.

Two months after the inoculation, tomato plants were harvested to examine gall formation on the wounded site. The disease incidence was calculated by dividing the number of plants with visible gall formation by the total number of inoculated plants. The sections with crown gall were cut out and sterilized in 35 mL of sterilization solution (3% NaOCl, 0.01% Tween 20) for 30 s, and then transferred to 35 mL of 70% ethanol and washed in 35 mL of sterile H_2_O three times (48). The sterilization was evaluated by spreading 100 μL of liquid from the third wash onto 523 agar plates and observing for 2 days to make sure there was no growth of bacteria. After sterilization, the crown galls were dissected from each of the plant segments, placed in a sterile petri dish, wrapped in sterile aluminum foil, frozen by liquid nitrogen, and stored at −80°C prior to DNA extraction.

### DNA extraction from crown galls.

Crown gall samples were homogenized with a pestle and mortar with liquid nitrogen. Then, 0.25 g of homogenized tissue was transferred to the beating tube of a DNeasy PowerSoil kit (Qiagen, Germany) following the manufacturer’s instructions. Concentrations of extracted DNA were determined using NanoDrop 1000 (Thermo Fisher Scientific, USA) and Qubit double-stranded DNA (dsDNA) high-sensitivity (HS) (Invitrogen, USA) systems.

### 16S rRNA gene amplification and sequencing.

Three rounds of amplicon sequencing were conducted in this study. Round I was conducted according to established protocols of soil microbiota studies using 16S rRNA gene variable regions (25). Each primer with a partial adaptor sequence was compatible with the Illumina TruSeq combinatorial dual index system. The first PCR amplifications were carried out in the following 25-μL reaction: 5 ng DNA template, 2× KAPA HiFi HotStart DNA polymerase ReadyMix (Roche, Switzerland), and 0.2 μM for each of the forward and reverse primers. The first PCR program and reaction details for V3 to V4 and V5- to V7 are listed in Table S4. The PCR products were purified using Ampure XP beads (Thermo Fisher Scientific, Inc., Sweden). The V5 to V7 PCR products underwent BluePippin (Sage Science) size-selection to remove tomato amplicon. To attach dual indices and full-length Illumina sequencing adapters, a second round of PCR amplifications was employed for 50-μL reactions with purified or size-selection PCR products, 2× KAPA HiFi HotStart DNA polymerase ReadyMix (Roche), and 0.5 μM for each of the forward and reverse primers. All of the library products of the second PCR were purified and pooled. The pooled libraries were loaded onto an Illumina MiSeq V3 flow cell (Illumina, USA) for 2 × 300-bp paired-end sequencing.

10.1128/mbio.00177-23.8 TABLE S4 Condition of 16S rRNA gene amplification. Download Table S4, DOCX file, 0.2 MB. Copyright © 2023 Wang et al. 2023 Wang et al. https://creativecommons.org/licenses/by/4.0/ This content is distributed under the terms of the Creative Commons Attribution 4.0 International license.

Round II of amplicon sequencing was conducted to test the effect of adding PCR blockers on reducing plant chloroplast and mitochondrial sequences. Three gall samples (1115-W21, 1115-W22, and 1115-W25) were used as the DNA templates to provide biological replicates. For each DNA sample, four primer pairs targeting different variable regions of 16S rRNA gene were used: V1 to V3, V3 to V4, V5 to V7, and V6 to V8 (Table S1A). Each primer contained a partial adaptor sequence of the Illumina TruSeq combinatorial dual index system. Cognate 3′ C_3_ spacer-modified oligonucleotides (blockers) which would block the amplification of tomato chloroplast and mitochondrial 16S rRNA genes were designed according to a previous study (22) and applied to one of the two PCRs for each sample-primer combination (Table S1B). The procedures for PCR and Illumina sequencing were based on those used for round I, but the amount of DNA template was increased to 25 ng to reduce PCR cycle time, and the annealing temperature was adjusted for blockers. More detailed technical information is provided in Table S4.

Based on results from the first two rounds of amplicon sequencing, an optimized protocol that utilizes blockers and targets the V5 to V7 region was used for round III. All 53 crown gall samples were included in this final round.

### Raw read processing and microbiota analysis.

The Illumina raw reads in FASTQ format were imported into QIIME 2 release 2020.8 (49). The primer region of imported sequences was trimmed using DADA2 (50). The denoised and merged paired-end reads were used to construct output feature tables containing amplicon sequence variant (ASVs) IDs and counts in different samples. For amplicon sequencing round III, the ASVs were further clustered into species-level OTUs based on 99% sequence identity using the qiime vsearch cluster-features-de-novo function (51), and the singletons were removed using the qiime feature-table filter-features function prior to the downstream analysis. The taxonomic information was inferred for representative OTU sequences via the pretrained q2-feature-classifier (52) and classify-sklearn naive Bayes classifier based on the SILVA version 138 non-redundant small subunit rRNA reference sequences dataset (SSU Ref NR 99) (53). The sequences assigned as chloroplasts, mitochondria, eukaryotes, archaea, or unknown were removed. After the filtering, a phylogenetic tree of eubacterial sequences was inferred using the q2-fragment-insertion plugin (54–57) based on the SILVA 128 SATé-Enabled Phylogenetic Placement (SEPP) reference database. Weighted UniFrac (58) analysis of those data sets was performed based on an insertion tree; the distance matrix and principal-coordinate analysis (PCoA) plot were generated using QIIME 2. For round III, phylogenic and diversity analysis was conducted using qiime diversity core-metrics-phylogenetic, and the subsampling depth was set to 9,800 reads/sample.

### Differential abundance analysis of OTU.

Analysis of the composition of microbiomes (ANCOM) was applied to identify OTUs that had significantly different relative abundance in the gallobiome associated with the C58 WT and T6SS mutants using the function qiime composition ancom in QIIME 2. The feature table collapsed into the family level was imported and transformed by centered log-ratio transformation. The null hypothesis of ANCOM is that the abundance of an OTU is not different between two study groups. After performing all comparisons between each OTU in two study groups, the times that the null hypothesis was rejected is called *w*. The OTUs having *w* values at the 70th percentile or higher are considered significant (59, 60).

### Isolation of *Sphingomonas* sp. R1.

*Sphingomonas* sp. R1 was isolated from a wounded tomato stem during a soil inoculation assay. After 10 dpi of soil inoculation as mentioned above, the 1-cm wounded segments of tomato seedlings were harvested and rinsed with sterile water and then transferred to 1 mL 0.9% NaCl. After vortexing at maximum speed for 3 min, the supernatant was transferred to a new microcentrifuge tube and spread on 523 agar plates via the exponential mode of the easySpiral Dilute system (reference [ref.] no. 414 000, Interscience, France). After growth at 25°C for 2 days, ~20 colonies were selected and streaked on 523 agar plates for pure culture. The partial 16S rRNA gene of the isolates was amplified and sequenced using the V5 to V7 primer set, which was used to conduct a BLAST search of the 16S rRNA gene sequence database in NCBI for identification of 16 bacterial species (Table S2).

### Interbacterial competition assay *in vitro* and *in planta*.

For interbacterial competition *in vitro*, the bacterial suspension was adjusted to an OD_600_ of 3.0 in 0.9% NaCl (wt/vol) after overnight culture. For the *in vitro* competition, the attacker strains (C58 WT, Δ*tssL*, and Δ*tssB*) were further diluted to an OD_600_ of 1, and the prey *Sphingomonas* sp. R1 containing pRL662::GFP was further diluted to an OD_600_ of 0.3 or 0.1 for mixing with the attacker in equal volumes for different density ratios. Two spots of 10 μL of mixture were spotted on *Agrobacterium* kill-triggering medium (21). After being coincubated for 16 h at 25°C, the colonies were scraped and resuspended in 1 mL 0.9% NaCl (wt/vol) and spread via the exponential mode of the easySpiral Dilute system (ref. no. 414 000, Interscience, France) on 523 medium containing 30 μg/mL gentamicin to recover the prey.

For agrobacterial colonization with or without *Sphingomonas* sp. R1, each of the agrobacterial strains (C58 WT, Δ*tssL*, and Δ*tssB*) with Gm^R^-GFP knock-in was mixed with *Sphingomonas* sp. R1 containing pBBR::mCherry at an OD_600_ of 1.0. The 5-μL mixture was inoculated on the needle-wounded stem of tomato seedlings with first pairs of true leaves. Tomato seedlings were harvested at 10 days postinoculation (dpi). For each plant, the 1-cm wounded segment was cut and rinsed with sterile water before being transferred into a sterilized Eppendorf tube containing 1 mL of 0.9% NaCl. After being vortexed at maximum speed for 3 min, the liquid was considered the rhizosphere sample. The samples were diluted and plated on medium with gentamicin for CFU calculation.

For counting the CFU of C58 and *Sphingomonas* sp. R1 in the tumor, the crown galls induced by direct inoculation (19) were collected after growing in the greenhouse for 3 weeks. The 1-cm tomato stem section containing the gall in middle was cut and homogenized with 1 mL 0.9% NaCl (wt/vol) using a sterilized mortar and pestle. The homogenized tissue was diluted 10-fold and spread via the exponential mode of the easySpiral Dilute system on 523 medium containing 50 ppm gentamicin to recover the attacker and prey. After incubation for 2 days at 25°C, the colonies were counted by using the Scan 500 automatic colony counter (ref. no. 436 000, Interscience, France; software version 8.6.1) to calculate CFU.

### Statistics and visualization.

GraphPad Prism 8 was used to perform *t* tests on sequencing read counts, one-way analysis of variance (ANOVA) on CFU counts, two-way ANOVA followed by Turkey’s multiple-comparison test on crown gall disease incidence, the alpha diversity index and Kruskal-Wallis test on tumor weight, and Spearman correlation on gall weight and the relative abundance of agrobacterial OTU. The alpha-rarefaction curves were plotted using phyloseq (61) in R version 3.6.2. ADONIS in the qiime diversity beta-group-significance function of QIIME 2 (62, 63) was used to test if groups of samples had significantly different microbiota compositions. The taxonomic composition plots were generated using qiime2R (version 0.99.35, https://github.com/jbisanz/qiime2R) in R studio 2.

### Data availability.

All raw data sets are available in the National Center for Biotechnology Information (NCBI) under BioProject accession number PRJNA894311.
