# Supplementary material for: Soil Inoculation and Blocker-Mediated Sequencing Show Effects of the Antibacterial T6SS on Agrobacterial Tumorigenesis and Gallobiome
Source: mBio. 2023 Mar 6;14(2):e00177-23. doi: 10.1128/mbio.00177-23 (PMC10128044; doi:10.1128/mbio.00177-23)
Supplement: TABLE S5 [file mbio.00177-23-s0009.docx]

Table S5. Metadata of harvested crown galls

|  |  |  |  |  | Harvest time | | |  |
| --- | --- | --- | --- | --- | --- | --- | --- | --- |
| #Sample ID | Strain* | Year of inoculation | Month of inoculation | Day of inoculation | Year | Month | Day | Weight (g) |
| 1115-11L | Δ*tssL* | 2018 | 11 | 15 | 2019 | 1 | 18 | 1.80 |
| 1115-12L^a^ | Δ*tssL* | 2018 | 11 | 15 | 2019 | 1 | 18 | 0.81 |
| 1115-13L^a^ | Δ*tssL* | 2018 | 11 | 15 | 2019 | 1 | 18 | 0.62 |
| 1115-14L^a^ | Δ*tssL* | 2018 | 11 | 15 | 2019 | 1 | 18 | 0.96 |
| 1115-15L | Δ*tssL* | 2018 | 11 | 15 | 2019 | 1 | 18 | 0.40 |
| 1115-16L | Δ*tssL* | 2018 | 11 | 15 | 2019 | 1 | 18 | 0.40 |
| 1115-17L | Δ*tssL* | 2018 | 11 | 15 | 2019 | 1 | 18 | 0.07 |
| 1115-18L | Δ*tssL* | 2018 | 11 | 15 | 2019 | 1 | 18 | 0.16 |
| 1115-19W | WT | 2018 | 11 | 15 | 2019 | 1 | 18 | 2.00 |
| 1115-20W | WT | 2018 | 11 | 15 | 2019 | 1 | 18 | 1.27 |
| 1115-21W^b^ | WT | 2018 | 11 | 15 | 2019 | 1 | 18 | 0.56 |
| 1115-22W^b^ | WT | 2018 | 11 | 15 | 2019 | 1 | 18 | 0.56 |
| 1115-23W | WT | 2018 | 11 | 15 | 2019 | 1 | 18 | 0.42 |
| 1115-24W | WT | 2018 | 11 | 15 | 2019 | 1 | 18 | 0.37 |
| 1115-25W^b^ | WT | 2018 | 11 | 15 | 2019 | 1 | 18 | 0.82 |
| 1115-26W | WT | 2018 | 11 | 15 | 2019 | 1 | 18 | 0.22 |
| 1115-27W | WT | 2018 | 11 | 15 | 2019 | 1 | 18 | 0.10 |
| 1115-28W | WT | 2018 | 11 | 15 | 2019 | 1 | 18 | 0.08 |
| 1115-29W | WT | 2018 | 11 | 15 | 2019 | 1 | 18 | 0.05 |
| 1115-30W | WT | 2018 | 11 | 15 | 2019 | 1 | 18 | 0.02 |
| 0704-1W | WT | 2019 | 7 | 4 | 2019 | 9 | 2 | 0.12 |
| 0704-2W | WT | 2019 | 7 | 4 | 2019 | 9 | 2 | 0.12 |
| 0704-3W | WT | 2019 | 7 | 4 | 2019 | 9 | 2 | 0.09 |
| 0704-4W | WT | 2019 | 7 | 4 | 2019 | 9 | 2 | 0.06 |
| 0704-5W | WT | 2019 | 7 | 4 | 2019 | 9 | 2 | 0.11 |
| 0704-6W | WT | 2019 | 7 | 4 | 2019 | 9 | 2 | 0.14 |
| 0704-7W | WT | 2019 | 7 | 4 | 2019 | 9 | 2 | 0.27 |
| 0704-1L | Δ*tssL* | 2019 | 7 | 4 | 2019 | 9 | 2 | 0.10 |
| 0704-2L | Δ*tssL* | 2019 | 7 | 4 | 2019 | 9 | 2 | 0.34 |
| 0704-3L | Δ*tssL* | 2019 | 7 | 4 | 2019 | 9 | 2 | 0.12 |
| 0704-4L | Δ*tssL* | 2019 | 7 | 4 | 2019 | 9 | 2 | 0.07 |
| 0704-5L | Δ*tssL* | 2019 | 7 | 4 | 2019 | 9 | 2 | 0.06 |
| 0704-6L | Δ*tssL* | 2019 | 7 | 4 | 2019 | 9 | 2 | 0.10 |
| 0704-7L | Δ*tssL* | 2019 | 7 | 4 | 2019 | 9 | 2 | 0.13 |
| 0704-1B | Δ*tssB* | 2019 | 7 | 4 | 2019 | 9 | 2 | 0.11 |
| 0704-2B | Δ*tssB* | 2019 | 7 | 4 | 2019 | 9 | 2 | 0.28 |
| 0704-3B | Δ*tssB* | 2019 | 7 | 4 | 2019 | 9 | 2 | 0.07 |
| 0704-4B | Δ*tssB* | 2019 | 7 | 4 | 2019 | 9 | 2 | 0.22 |
| 0704-5B | Δ*tssB* | 2019 | 7 | 4 | 2019 | 9 | 2 | 0.06 |
| 0722-1W | WT | 2019 | 7 | 22 | 2019 | 9 | 28 | 0.03 |
| 0722-2W | WT | 2019 | 7 | 22 | 2019 | 9 | 28 | 0.04 |
| 0722-3W | WT | 2019 | 7 | 22 | 2019 | 9 | 28 | 0.07 |
| 0722-4W | WT | 2019 | 7 | 22 | 2019 | 9 | 28 | 0.05 |
| 0722-5W | WT | 2019 | 7 | 22 | 2019 | 9 | 28 | 0.13 |
| 0722-6W | WT | 2019 | 7 | 22 | 2019 | 9 | 28 | 0.05 |
| 0722-1B | Δ*tssB* | 2019 | 7 | 22 | 2019 | 9 | 28 | 0.01 |
| 0722-2B | Δ*tssB* | 2019 | 7 | 22 | 2019 | 9 | 28 | 0.01 |
| 0729-1W | WT | 2019 | 7 | 29 | 2019 | 9 | 29 | 0.03 |
| 0729-2W | WT | 2019 | 7 | 29 | 2019 | 9 | 29 | 0.05 |
| 0729-3W | WT | 2019 | 7 | 29 | 2019 | 9 | 29 | 0.04 |
| 0729-4W | WT | 2019 | 7 | 29 | 2019 | 9 | 29 | 0.05 |
| 0729-5W | WT | 2019 | 7 | 29 | 2019 | 9 | 29 | 0.04 |
| 0729-6W | WT | 2019 | 7 | 29 | 2019 | 9 | 29 | 0.01 |
| 0729-7W | WT | 2019 | 7 | 29 | 2019 | 9 | 29 | 0.05 |
| 0729-8W | WT | 2019 | 7 | 29 | 2019 | 9 | 29 | 0.08 |
| 0729-9W | WT | 2019 | 7 | 29 | 2019 | 9 | 29 | 0.03 |
| 0729-1L | Δ*tssL* | 2019 | 7 | 29 | 2019 | 9 | 29 | 0.01 |
| 0729-2L | Δ*tssL* | 2019 | 7 | 29 | 2019 | 9 | 29 | 0.05 |
| 0729-3L | Δ*tssL* | 2019 | 7 | 29 | 2019 | 9 | 29 | 0.08 |
| 0729-4L | Δ*tssL* | 2019 | 7 | 29 | 2019 | 9 | 29 | 0.03 |
| 0729-1B | ΔtssB | 2019 | 7 | 29 | 2019 | 9 | 29 | 0.09 |
| 0729-2B | ΔtssB | 2019 | 7 | 29 | 2019 | 9 | 29 | 0.07 |
| 1005-1W | WT | 2019 | 10 | 5 | 2019 | 12 | 13 | 0.27 |
| 1005-2W | WT | 2019 | 10 | 5 | 2019 | 12 | 13 | 0.16 |
| 1005-3W | WT | 2019 | 10 | 5 | 2019 | 12 | 13 | 0.09 |
| 1005-4W | WT | 2019 | 10 | 5 | 2019 | 12 | 13 | 0.04 |
| 1005-5W | WT | 2019 | 10 | 5 | 2019 | 12 | 13 | 0.01 |
| 1005-6W | WT | 2019 | 10 | 5 | 2019 | 12 | 13 | 0.09 |
| 1005-7W | WT | 2019 | 10 | 5 | 2019 | 12 | 13 | 0.01 |
| 1005-1L | Δ*tssL* | 2019 | 10 | 5 | 2019 | 12 | 13 | 0.23 |
| 1005-2L | Δ*tssL* | 2019 | 10 | 5 | 2019 | 12 | 13 | 0.32 |
| 1005-3L | Δ*tssL* | 2019 | 10 | 5 | 2019 | 12 | 13 | 0.09 |
| 1005-4L | Δ*tssL* | 2019 | 10 | 5 | 2019 | 12 | 13 | 0.04 |
| 1005-5L | Δ*tssL* | 2019 | 10 | 5 | 2019 | 12 | 13 | 0.01 |
| 1005-1B | Δ*tssB* | 2019 | 10 | 5 | 2019 | 12 | 13 | 0.23 |
| 1005-2B | Δ*tssB* | 2019 | 10 | 5 | 2019 | 12 | 13 | 0.56 |
| 1005-3B | Δ*tssB* | 2019 | 10 | 5 | 2019 | 12 | 13 | 0.16 |
| 1005-4B | Δ*tssB* | 2019 | 10 | 5 | 2019 | 12 | 13 | 0.05 |
| 1005-5B | Δ*tssB* | 2019 | 10 | 5 | 2019 | 12 | 13 | 0.06 |
| 1005-6B | Δ*tssB* | 2019 | 10 | 5 | 2019 | 12 | 13 | 0.03 |
| 1005-7B | Δ*tssB* | 2019 | 10 | 5 | 2019 | 12 | 13 | 0.06 |
| 1030-1W | WT | 2019 | 10 | 30 | 2020 | 1 | 9 | 0.34 |
| 1030-2W | WT | 2019 | 10 | 30 | 2020 | 1 | 9 | 0.12 |
| 1030-3W | WT | 2019 | 10 | 30 | 2020 | 1 | 9 | 0.05 |
| 1030-4W | WT | 2019 | 10 | 30 | 2020 | 1 | 9 | 0.03 |
| 1030-5W | WT | 2019 | 10 | 30 | 2020 | 1 | 9 | 0.05 |
| 1030-6W | WT | 2019 | 10 | 30 | 2020 | 1 | 9 | 0.11 |
| 1030-7W | WT | 2019 | 10 | 30 | 2020 | 1 | 9 | 0.02 |
| 1030-8W | WT | 2019 | 10 | 30 | 2020 | 1 | 9 | 0.01 |
| 1030-1L | Δ*tssL* | 2019 | 10 | 30 | 2020 | 1 | 9 | 0.50 |
| 1030-2L | Δ*tssL* | 2019 | 10 | 30 | 2020 | 1 | 9 | 0.07 |
| 1030-3L | Δ*tssL* | 2019 | 10 | 30 | 2020 | 1 | 9 | 0.03 |
| 1030-4L | Δ*tssL* | 2019 | 10 | 30 | 2020 | 1 | 9 | 0.01 |
| 1030-1B | Δ*tssB* | 2019 | 10 | 30 | 2020 | 1 | 9 | 0.17 |
| 1030-2B | Δ*tssB* | 2019 | 10 | 30 | 2020 | 1 | 9 | 0.06 |
| 1030-3B | Δ*tssB* | 2019 | 10 | 30 | 2020 | 1 | 9 | 0.08 |
| 1030-4B | Δ*tssB* | 2019 | 10 | 30 | 2020 | 1 | 9 | 0.00 |
| 1030-5B | Δ*tssB* | 2019 | 10 | 30 | 2020 | 1 | 9 | 0.02 |
| 1030-6B | Δ*tssB* | 2019 | 10 | 30 | 2020 | 1 | 9 | 0.01 |
| 1111-1W | WT | 2019 | 11 | 11 | 2020 | 1 | 14 | 0.26 |
| 1111-2W | WT | 2019 | 11 | 11 | 2020 | 1 | 14 | 0.19 |
| 1111-2.1W | WT | 2019 | 11 | 11 | 2020 | 1 | 14 | 0.04 |
| 1111-3W | WT | 2019 | 11 | 11 | 2020 | 1 | 14 | 0.25 |
| 1111-3.1W | WT | 2019 | 11 | 11 | 2020 | 1 | 14 | 0.04 |
| 1111-4W | WT | 2019 | 11 | 11 | 2020 | 1 | 14 | 0.13 |
| 1111-5W | WT | 2019 | 11 | 11 | 2020 | 1 | 14 | 0.01 |
| 1111-6W | WT | 2019 | 11 | 11 | 2020 | 1 | 14 | 0.04 |
| 1111-7W | WT | 2019 | 11 | 11 | 2020 | 1 | 14 | 0.05 |
| 1111-8W | WT | 2019 | 11 | 11 | 2020 | 1 | 14 | 0.11 |
| 1111-8.1W | WT | 2019 | 11 | 11 | 2020 | 1 | 14 | 0.09 |
| 1111-8.2W | WT | 2019 | 11 | 11 | 2020 | 1 | 14 | 0.09 |
| 1111-9W | WT | 2019 | 11 | 11 | 2020 | 1 | 14 | 0.02 |
| 1111-10W | WT | 2019 | 11 | 11 | 2020 | 1 | 14 | 0.07 |
| 1111-11W | WT | 2019 | 11 | 11 | 2020 | 1 | 14 | 0.02 |
| 1111-12W | WT | 2019 | 11 | 11 | 2020 | 1 | 14 | 0.04 |
| 1111-13W | WT | 2019 | 11 | 11 | 2020 | 1 | 14 | 0.27 |
| 1111-13.1W | WT | 2019 | 11 | 11 | 2020 | 1 | 14 | 0.18 |
| 1111-13.2W | WT | 2019 | 11 | 11 | 2020 | 1 | 14 | 0.12 |
| 1111-14W | WT | 2019 | 11 | 11 | 2020 | 1 | 14 | 0.04 |
| 1111-15W | WT | 2019 | 11 | 11 | 2020 | 1 | 14 | 0.01 |
| 1111-1L | Δ*tssL* | 2019 | 11 | 11 | 2020 | 1 | 14 | 0.21 |
| 1111-2L | Δ*tssL* | 2019 | 11 | 11 | 2020 | 1 | 14 | 0.04 |
| 1111-2.1L | Δ*tssL* | 2019 | 11 | 11 | 2020 | 1 | 14 | 0.02 |
| 1111-3L | Δ*tssL* | 2019 | 11 | 11 | 2020 | 1 | 14 | 0.07 |
| 1111-3.1L | Δ*tssL* | 2019 | 11 | 11 | 2020 | 1 | 14 | 0.03 |
| 1111-4L | Δ*tssL* | 2019 | 11 | 11 | 2020 | 1 | 14 | 0.03 |
| 1111-5L | Δ*tssL* | 2019 | 11 | 11 | 2020 | 1 | 14 | 0.08 |
| 1111-6L | Δ*tssL* | 2019 | 11 | 11 | 2020 | 1 | 14 | 0.08 |
| 1111-7L | Δ*tssL* | 2019 | 11 | 11 | 2020 | 1 | 14 | 0.14 |
| 1111-8L | Δ*tssL* | 2019 | 11 | 11 | 2020 | 1 | 14 | 0.01 |
| 1111-9L | Δ*tssL* | 2019 | 11 | 11 | 2020 | 1 | 14 | 0.03 |
| 1111-1B | Δ*tssB* | 2019 | 11 | 11 | 2020 | 1 | 14 | 0.18 |
| 1111-2B | Δ*tssB* | 2019 | 11 | 11 | 2020 | 1 | 14 | 0.09 |
| 1111-3B | Δ*tssB* | 2019 | 11 | 11 | 2020 | 1 | 14 | 0.03 |
| 1111-4B | Δ*tssB* | 2019 | 11 | 11 | 2020 | 1 | 14 | 0.03 |
| 1111-5B | Δ*tssB* | 2019 | 11 | 11 | 2020 | 1 | 14 | 0.02 |
| 1111-6B | Δ*tssB* | 2019 | 11 | 11 | 2020 | 1 | 14 | 0.03 |
| 1111-7B | Δ*tssB* | 2019 | 11 | 11 | 2020 | 1 | 14 | 0.01 |
| 1111-8B | Δ*tssB* | 2019 | 11 | 11 | 2020 | 1 | 14 | 0.01 |

** Agrobacterium* C58, ^a^ Used in Round I sequencing, ^b^ Used in both Round I and II sequencing.
