# Supplementary material for: Soil Inoculation and Blocker-Mediated Sequencing Show Effects of the Antibacterial T6SS on Agrobacterial Tumorigenesis and Gallobiome
Source: mBio. 2023 Mar 6;14(2):e00177-23. doi: 10.1128/mbio.00177-23 (PMC10128044; doi:10.1128/mbio.00177-23)
Supplement: TABLE S1 [file mbio.00177-23-s0005.docx]

Table S1A. 16S rRNA gene primer sets used in this study

| Targeted Region | Name | Sequence (5’-3’) | Size of bacterial amplicons (bp) | Size of tomato amplicons (bp)* |
| --- | --- | --- | --- | --- |
| Round I |  |  |  |  |
| V3-V4 | 341F | CCTACGGGNGGCWGCAG | 444 | 396 (cp); 395 (mt) |
|  | 785R | GACTACHVGGGTATCTAATCC |  |  |
| V5-V7 | 799F | AACMGGATTAGATACCCKG | 394 | 376(cp); 742(mt) |
|  | 1193R | ACGTCATCCCCACCTTCC |  |  |
| Round II |  |  |  |  |
| V1-V3 | 68F | TNANACATGCAAGTCGRRCG | 425 | 440 (cp); 474(mt) |
|  | 518R | WTTACCGCGGCTGCTGG |  |  |
| V3-V4 | 341F | CCTACGGGNGGCWGCAG | 429 | 408 (cp); 391(mt) |
|  | 688R | CGCTTTCGHDCCTCAGYGTCA | 405 |  |
| V5-V7 | 819F | GTCCACVCCSTAAACGWTG | 553 | 551 (cp); 915(mt) |
|  | 1276R | RCGATTACTAGCGAHTCC |  |  |
| V6-V8 | 895F | CRCCTGGGGAGTRCRG | 530 | 527 (cp); 894 (mt) |
|  | 1391R | GACGGGCGGTGTGTRCA |  |  |

*cp, chloroplast; mt, mitochondrion

Table S1B. Corresponding blockers (3’ modified oligonucleotides with C3 spacer)

| Targeted Region | Name* | Sequence | Tm (℃) |
| --- | --- | --- | --- |
| V1 | 68f_blocker_mt | GTCGAACGTTGTTTTCGGGGAG | 62 |
| V1 | 68f_blocker_cp | GTCGGACGGGAAACACG | 61 |
| V4 | 688ra_blocker_mt | CGTCGGTAGGGACCCAGAGAGCT | 69 |
| V4 | 688ra_blocker_cp | TGTCAGTGTCGGCCCAGCAGAGT | 69 |
| V5 | 819f_blocker_mt | ACGATGAGTGTTCGCCCTTG | 61 |
| V5 | 819f_blocker_cp | AAACGATGGATACTAGGTGCTGT | 60 |
| V6 | 895f_blocker_mt | AGTACGGTCGCAAGACCG | 61 |
| V6 | 895f_blocker_cp | GGAGTACGTTCGCAAGAATG | 60 |

^*^ cp, chloroplast; mt, mitochondrion
