# Supplementary material for: Soil Inoculation and Blocker-Mediated Sequencing Show Effects of the Antibacterial T6SS on Agrobacterial Tumorigenesis and Gallobiome
Source: mBio. 2023 Mar 6;14(2):e00177-23. doi: 10.1128/mbio.00177-23 (PMC10128044; doi:10.1128/mbio.00177-23)
Supplement: TABLE S6 [file mbio.00177-23-s0010.docx]

Table S6. Crown gall metadata used for analysis of amplicon sequencing round III

| #Sample ID | Strain | Year_of_  inoculation | Month_of_  inoculation | Weight | Season | Trial | Batch |
| --- | --- | --- | --- | --- | --- | --- | --- |
| #q2:types | category | category | category | numeric | category | category | category |
| 1115-L12 | Δ*tssL* | 2018 | Nov | 0.81 | Winter | 181115 | Batch 1 |
| 1115-L13 | Δ*tssL* | 2018 | Nov | 0.62 | Winter | 181115 | Batch 1 |
| 1115-L14 | Δ*tssL* | 2018 | Nov | 0.96 | Winter | 181115 | Batch 1 |
| 1115-W21 | WT | 2018 | Nov | 0.56 | Winter | 181115 | Batch 1 |
| 1115-W22 | WT | 2018 | Nov | 0.56 | Winter | 181115 | Batch 1 |
| 1115-W25 | WT | 2018 | Nov | 0.82 | Winter | 181115 | Batch 1 |
| 0704-W1 | WT | 2019 | Jul | 0.12 | Summer | 190704 | Batch 4 |
| 0704-W2 | WT | 2019 | Jul | 0.12 | Summer | 190704 | Batch 4 |
| 0704-W3 | WT | 2019 | Jul | 0.09 | Summer | 190704 | Batch 4 |
| 0704-W4 | WT | 2019 | Jul | 0.06 | Summer | 190704 | Batch 4 |
| 0704-W5 | WT | 2019 | Jul | 0.11 | Summer | 190704 | Batch 4 |
| 0704-W6 | WT | 2019 | Jul | 0.14 | Summer | 190704 | Batch 4 |
| 0704-W7 | WT | 2019 | Jul | 0.27 | Summer | 190704 | Batch 4 |
| 0704-L1 | Δ*tssL* | 2019 | Jul | 0.1 | Summer | 190704 | Batch 4 |
| 0704-L2 | Δ*tssL* | 2019 | Jul | 0.34 | Summer | 190704 | Batch 4 |
| 0704-L3 | Δ*tssL* | 2019 | Jul | 0.12 | Summer | 190704 | Batch 4 |
| 0704-B1 | Δ*tssB* | 2019 | Jul | 0.11 | Summer | 190704 | Batch 4 |
| 0704-B2 | Δ*tssB* | 2019 | Jul | 0.28 | Summer | 190704 | Batch 4 |
| 0704-B3 | Δ*tssB* | 2019 | Jul | 0.07 | Summer | 190704 | Batch 4 |
| 1005-W1 | WT | 2019 | Oct | 0.27 | Summer | 191005 | Batch 7 |
| 1005-W2 | WT | 2019 | Oct | 0.16 | Summer | 191005 | Batch 7 |
| 1005-W3 | WT | 2019 | Oct | 0.09 | Summer | 191005 | Batch 7 |
| 1005-W6 | WT | 2019 | Oct | 0.09 | Summer | 191005 | Batch 7 |
| 1005-L1 | Δ*tssL* | 2019 | Oct | 0.23 | Summer | 191005 | Batch 7 |
| 1005-L2 | Δ*tssL* | 2019 | Oct | 0.32 | Summer | 191005 | Batch 7 |
| 1005-L3 | Δ*tssL* | 2019 | Oct | 0.09 | Summer | 191005 | Batch 7 |
| 1005-B1 | Δ*tssB* | 2019 | Oct | 0.23 | Summer | 191005 | Batch 7 |
| 1005-B2 | Δ*tssB* | 2019 | Oct | 0.56 | Summer | 191005 | Batch 7 |
| 1005-B3 | Δ*tssB* | 2019 | Oct | 0.16 | Summer | 191005 | Batch 7 |
| 1005-B5 | Δ*tssB* | 2019 | Oct | 0.06 | Summer | 191005 | Batch 7 |
| 1005-B7 | Δ*tssB* | 2019 | Oct | 0.06 | Summer | 191005 | Batch 7 |
| 1030-W1 | WT | 2019 | Oct | 0.34 | Winter | 191030 | Batch 8 |
| 1030-W2 | WT | 2019 | Oct | 0.12 | Winter | 191030 | Batch 8 |
| 1030-W6 | WT | 2019 | Oct | 0.11 | Winter | 191030 | Batch 8 |
| 1030-L1 | Δ*tssL* | 2019 | Oct | 0.5 | Winter | 191030 | Batch 8 |
| 1030-L2 | Δ*tssL* | 2019 | Oct | 0.07 | Winter | 191030 | Batch 8 |
| 1030-B1 | Δ*tssL* | 2019 | Oct | 0.17 | Winter | 191030 | Batch 8 |
| 1030-B2 | Δ*tssL* | 2019 | Oct | 0.06 | Winter | 191030 | Batch 8 |
| 1030-B3 | Δ*tssL* | 2019 | Oct | 0.08 | Winter | 191030 | Batch 8 |
| 1111-W1 | WT | 2019 | Nov | 0.26 | Winter | 191111 | Batch 9 |
| 1111-W2 | WT | 2019 | Nov | 0.19 | Winter | 191111 | Batch 9 |
| 1111-W3 | WT | 2019 | Nov | 0.25 | Winter | 191111 | Batch 9 |
| 1111-W4 | WT | 2019 | Nov | 0.13 | Winter | 191111 | Batch 9 |
| 1111-W8 | WT | 2019 | Nov | 0.11 | Winter | 191111 | Batch 9 |
| 1111-W10 | WT | 2019 | Nov | 0.07 | Winter | 191111 | Batch 9 |
| 1111-W13 | WT | 2019 | Nov | 0.27 | Winter | 191111 | Batch 9 |
| 1111-L1 | Δ*tssL* | 2019 | Nov | 0.21 | Winter | 191111 | Batch 9 |
| 1111-L3 | Δ*tssL* | 2019 | Nov | 0.07 | Winter | 191111 | Batch 9 |
| 1111-L5 | Δ*tssL* | 2019 | Nov | 0.08 | Winter | 191111 | Batch 9 |
| 1111-L6 | Δ*tssL* | 2019 | Nov | 0.08 | Winter | 191111 | Batch 9 |
| 1111-L7 | Δ*tssL* | 2019 | Nov | 0.14 | Winter | 191111 | Batch 9 |
| 1111-B1 | Δ*tssB* | 2019 | Nov | 0.18 | Winter | 191111 | Batch 9 |
| 1111-B2 | Δ*tssB* | 2019 | Nov | 0.09 | Winter | 191111 | Batch 9 |
