# Supplementary figures and images for: Soil Inoculation and Blocker-Mediated Sequencing Show Effects of the Antibacterial T6SS on Agrobacterial Tumorigenesis and Gallobiome
Source: mBio. 2023 Mar 6;14(2):e00177-23. doi: 10.1128/mbio.00177-23 (PMC10128044; doi:10.1128/mbio.00177-23)

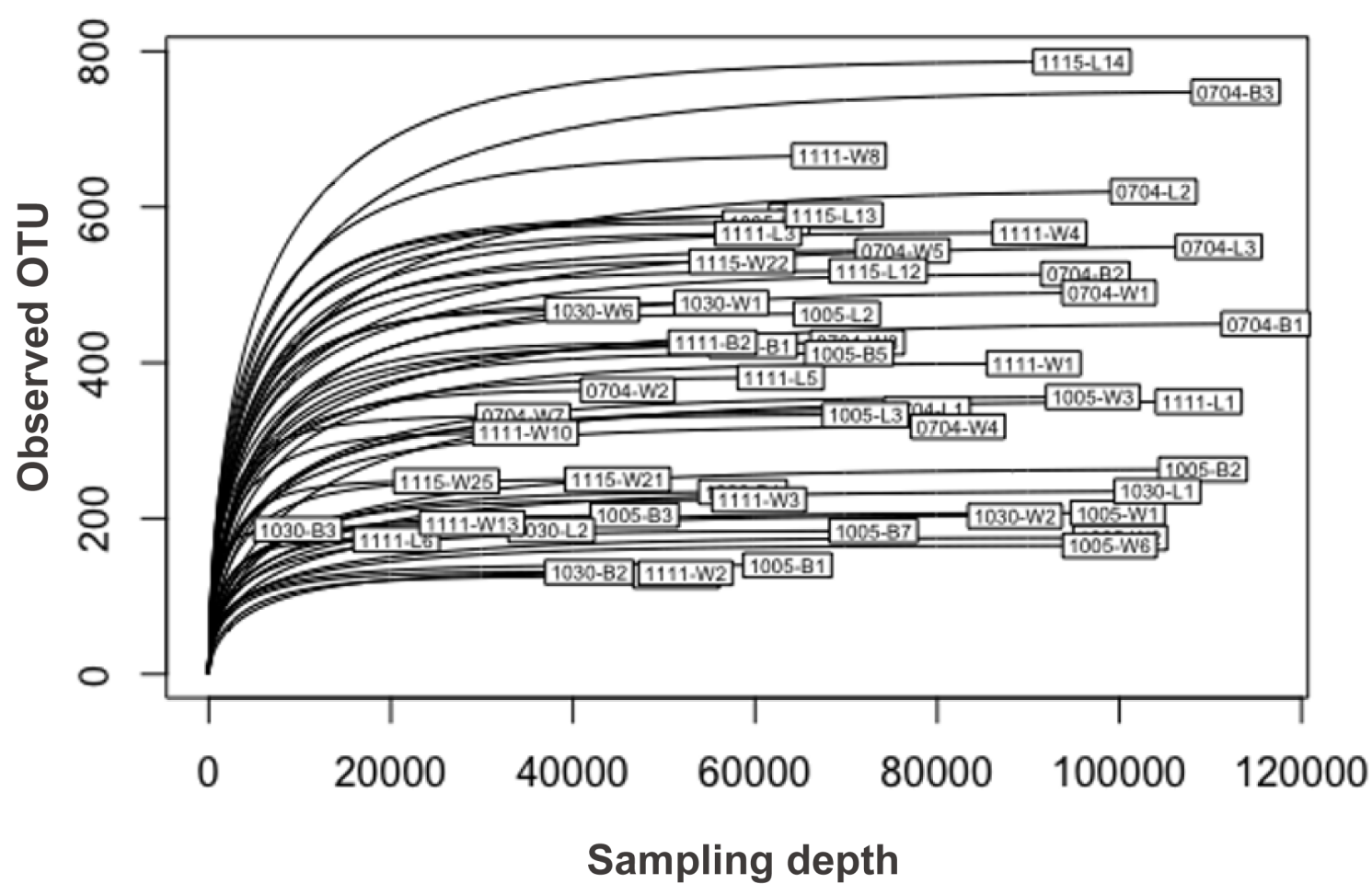

Supplement: FIG S2 [file mbio.00177-23-s0002.pdf]

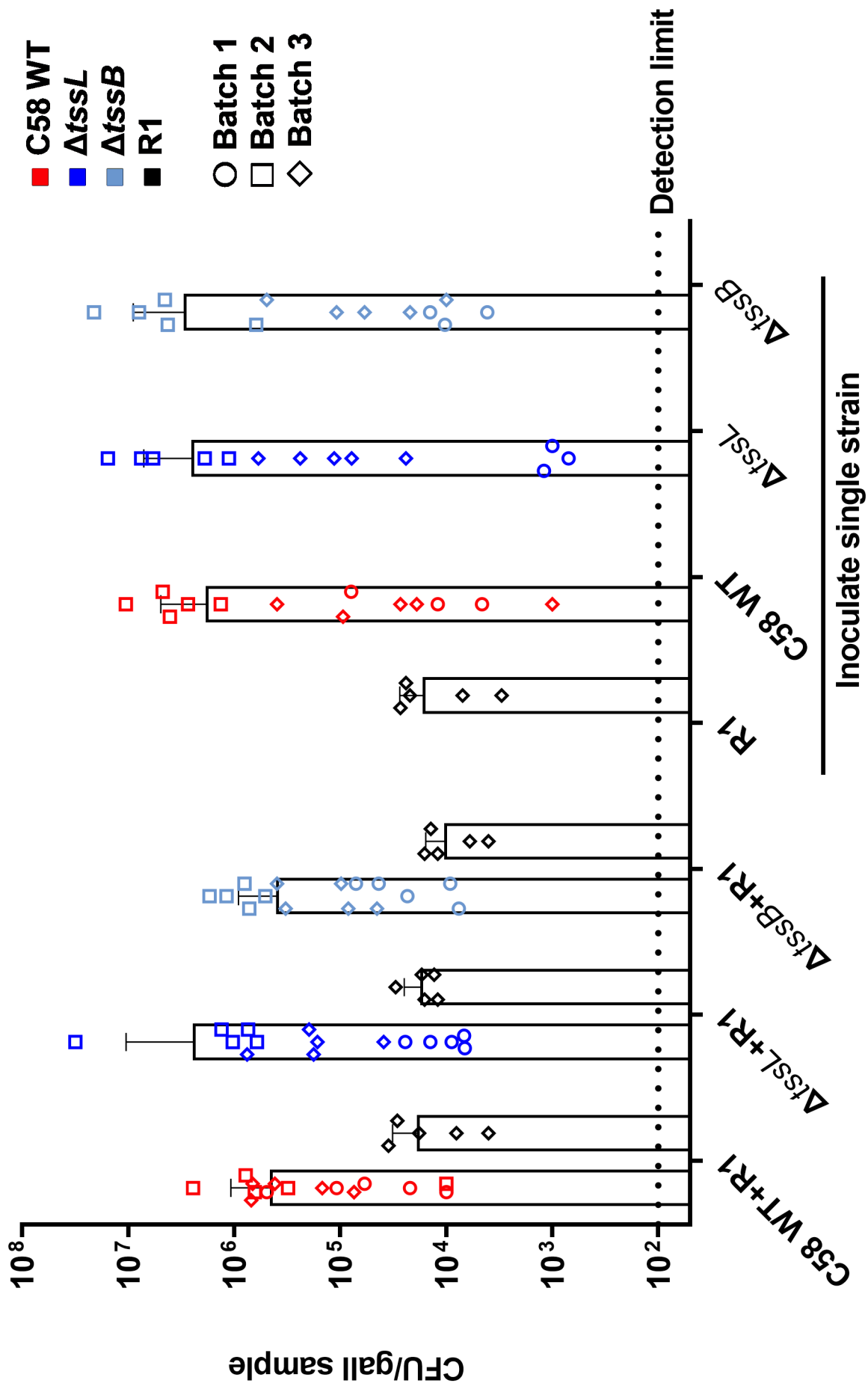

Supplement: FIG S3 [file mbio.00177-23-s0003.pdf]

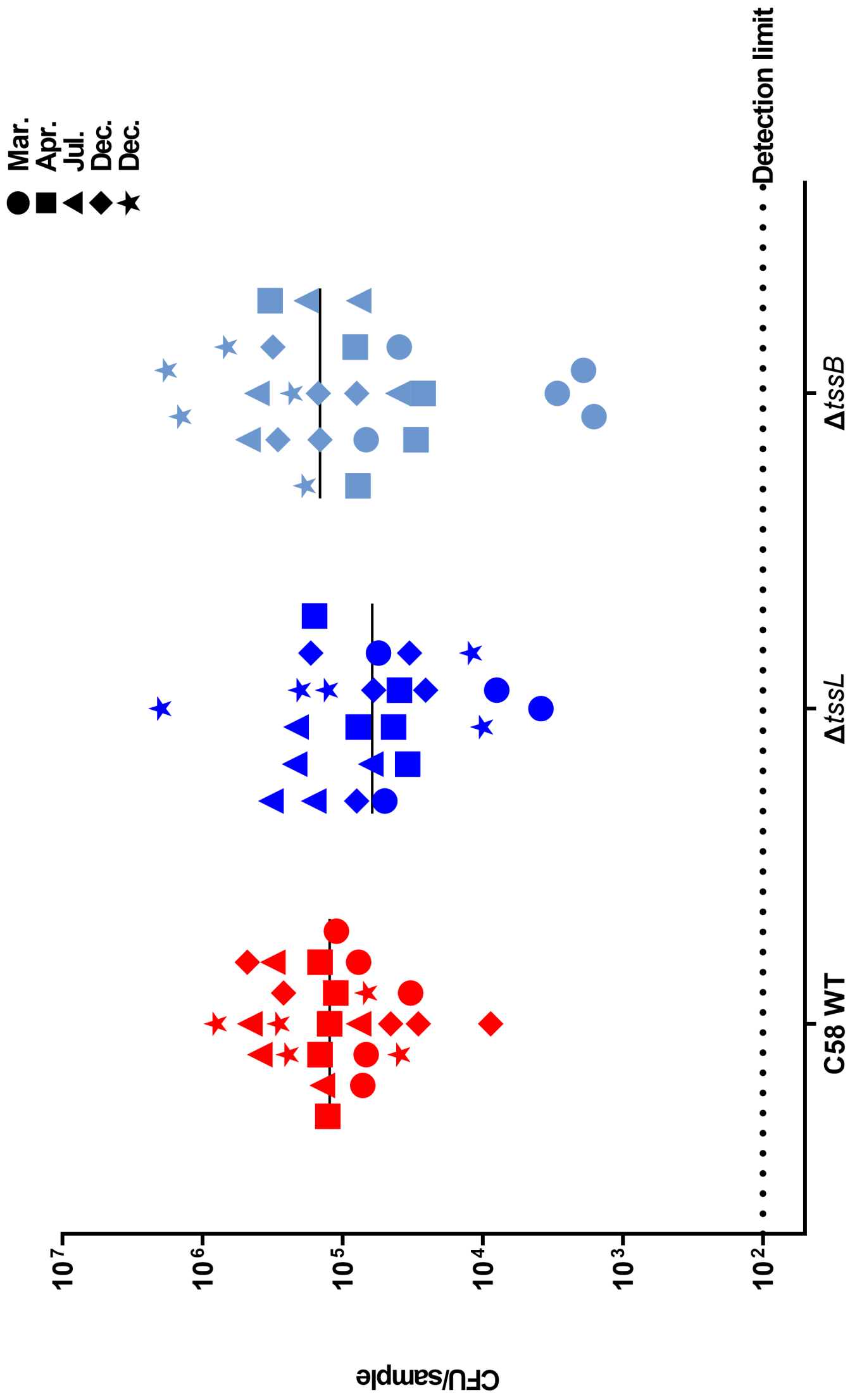

Supplement: FIG S4 [file mbio.00177-23-s0004.pdf]

Table S4. Condition of 16S rRNA gene amplification


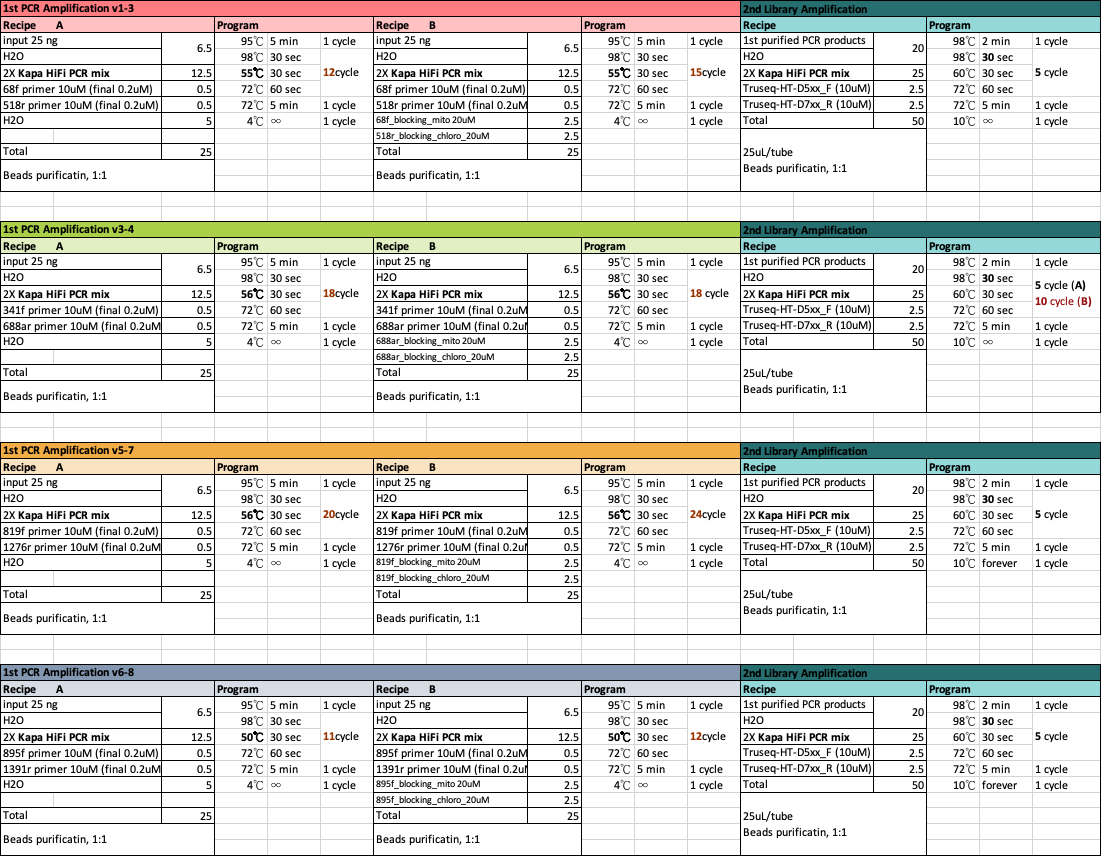

Supplement: TABLE S4 [file mbio.00177-23-s0008.docx]
