# Supplementary material for: Soil Inoculation and Blocker-Mediated Sequencing Show Effects of the Antibacterial T6SS on Agrobacterial Tumorigenesis and Gallobiome
Source: mBio. 2023 Mar 6;14(2):e00177-23. doi: 10.1128/mbio.00177-23 (PMC10128044; doi:10.1128/mbio.00177-23)
Supplement: TABLE S2 [file mbio.00177-23-s0006.docx]

Table S2. Identification of bacterial isolates from tomato rhizosphere

| Isolates | Genus | Per. Identity* |
| --- | --- | --- |
| R1 | *Sphingomonas* sp. | 100.00% |
| R3 | *Rhizobium* sp. | 99.43% |
| R4 | *Pseudacidovorax* sp. | 99.81% |
| R5 | *Roseateles* sp. | 100.00% |
| R6 | *Pseudacidovorax* sp. | 99.81% |
| R7 | *Flavobacterium* sp. | 98.86% |
| BR3-1 | *Pseudomonas* sp. | 96.88% |
| BR3-2 | *Acinetobacter* sp. | 99.78% |
| BR3-3 | *Pseudomonas* sp. | 96.98% |
| CKR3-4 | *Pseudomonas* sp. | 97.42% |
| CKR3-5 | *Microbacterium* sp. | 100.00% |
| CKR3-6 | *Pseudomonas* sp. | 99.56% |
| BE3-7 | *Microbacterium* sp. | 100.00% |
| BE3-8 | *Microbacterium* sp. | 100.00% |
| BE3-9 | *Microbacterium* sp. | 100.00% |
| BE5-10 | *Microbacterium* sp. | 100.00% |

* The partial 16S rRNA genes were amplified by V5-V7 primer set and the sequences were blast against 16S ribosomal RNA sequences database in NCBI.
