# Supplementary material for: Soil Inoculation and Blocker-Mediated Sequencing Show Effects of the Antibacterial T6SS on Agrobacterial Tumorigenesis and Gallobiome
Source: mBio. 2023 Mar 6;14(2):e00177-23. doi: 10.1128/mbio.00177-23 (PMC10128044; doi:10.1128/mbio.00177-23)
Supplement: TABLE S3 [file mbio.00177-23-s0007.docx]

Table S3A. Bacterial strains and plasmids used in this study

| Strain/plasmid | Characteristics | EML No. | Reference/ Source |
| --- | --- | --- | --- |
| *Escherichia coli* DH10B | Host for DNA cloning | 455 | Invitrogen |
| *Agrobacterium* C58 | Wild-type, virulent strain containing nonpaline-type Ti-plasimid, pTiC58 | 530 | Eugene Nester [1] |
|  | Δ*tssL*, deletion mutant of *tssL* gene (Atu4333) | 1073 | [2] |
|  | Δ*tssB*, deletion mutant of *tssB* gene (Atu4342) | 1109 | [2] |
|  | Gm^R^-GFP knocked-in chromosome | 4094 | This study |
|  | Δ*tssL*, Gm^R^-GFP knocked-in chromosome | 4097 | This study |
|  | Δ*tssB*, Gm^R^-GFP knocked-in chromosome | 4100 | This study |
| *Sphingomonas sp.* R1 | Wild type, isolated from tomato rhizosphere | 4107 | This study |
| pJQ-COM | Gm^R^, for generating DNA knock-in adjacent to gene *ActC* in C58 | 2236 | [3] |
| pJQ-com-Gm^r^ GFP | Gm^R^, for generate Gm^R^ and GFP knocked-in C58 | 4091 | This study |
| pRL662∷GFP (S65T) | Gm^R^, constitutively expressing GFP (S56T) | 3375 | This study |
| pBBR1MCS2-mCherry | Km^R^, constitutively expressing mCherry | 3022 | Stanton B. Gelvin |

**References**

1. Wood, D.W., et al., The genome of the natural genetic engineer *Agrobacterium tumefaciens* C58. Science, 2001. **294**(5550): p. 2317-23.

2. Lin, J.S., L.S. Ma, and E.M. Lai, Systematic Dissection of the *Agrobacterium* Type VI Secretion System Reveals Machinery and Secreted Components for Subcomplex Formation. PLoS One, 2013. **8**(7): p. e67647.

3. Liu, A.C., et al., A citrate-inducible gene, encoding a putative tricarboxylate transporter, is downregulated by the organic solvent DMSO in *Agrobacterium tumefaciens*. J Appl Microbiol, 2008. **105**(5): p. 1372-1383.

Table S3B. Primers used for plasmid construction

| Name | Sequence | Reference |
| --- | --- | --- |
| BclI-GFP-GmR-F-25 | AAAAATGATCAGTGAGCGCGCGTAATA CGACTCAC | This study |
| BclI-GFP-GmR-R-25 | AAAAATGATCAGGGTACCGAGCTCGAA TTGACATAAG | This study |
| 3'sacB-5'GmR pJQ200 F | TGCGCCAAGCTTCCTGCTGAACATC | This study |
| 3'sacB-5'GmR pJQ200 R | TTGAGCAGCCGCGTAGTGAGATCTA | This study |
